# Cultivar-Specific Phenolic and Fatty-Acid Composition of Albanian Monovarietal Olive Oils

**DOI:** 10.3390/foods15203726

**Published:** 2026-10-19

**Authors:** Fatjon Hoxha, Mivan Peçi, Concepción Muñoz Díez, Feliciano Priego Capote, Adhurim Lazaj, Anila Kopali, Elisa Peçi, Onejda Kyçyk, Hristofor Miho

**Affiliations:** 1 Department of Agri—Food Technology, Faculty of Biotechnology and Food, Agricultural University of Tirana, Rruga Paisi Vodica, 1025 Tirana, Albania; fhoxha@ubt.edu.al (F.H.);; 2 Department of Social Sciences and Law, University of Jaen, Campus Las Lagunillas, 23071 Jaen, Spain; 3 Department of Agronomy, University of Cordoba, Campus de Rabanales, 14071 Córdoba, Spain; 4 Department of Analytical Chemistry, University of Cordoba, Campus de Rabanales, 14071 Córdoba, Spain; 5 Agricultural Technology Transfer Center (ATTC) of Vlorë, 9411 Vlorë, Albania; 6 Department of Food Microbiology, Water Monitoring and Quality, Food Safety and Veterinary Institute (ISUV), Rruga Aleksandër Moisiu 82, 1005 Tirana, Albania; 7 Food Research Centre, Faculty of Biotechnology and Food, Agricultural University of Tirana, Rruga Paisi Vodica, 1025 Tirana, Albania

**Keywords:** Albanian olive germplasm, *Olea europaea* L., virgin olive oil, phenolic compounds, fatty acids, oleocanthal, oleic acid, oil yield

## Abstract

Albanian olive genetic resources remain under-characterised despite their potential for differentiated oil production. We evaluated 11 authenticated cultivars conserved in the Vlorë germplasm collection under common field, maturity and standardised industrial three-phase extraction conditions. Phenolics profile of those cultivars was quantified by liquid chromatography–tandem mass spectrometry (LC-MS/MS), and fatty-acid composition was determined by gas chromatography with flame-ionisation detection (GC-FID). Also, fruit moisture and oil content were analysed in maturity index 3. All these analytical parameters were integrated through univariate and multivariate statistical analyses. Cultivar factor significantly affected every trait. Total quantified phenolics ranged from 296.5 mg kg^−1^ in ‘U.K. Berat’ to 791.0 mg kg^−1^ in ‘Managjel FV’; the latter was oleocanthal-rich, whereas ‘U.H. Himare’, ‘Pulazeqin’, ‘Ulliri i Kuq’ and ‘Kotruvsi’ showed distinctive oleacein-, oleuropein-aglycone/oleomissional-, ligstroside-aglycone/tyrosol-, and luteolin/hydroxytyrosol-rich profiles, respectively. Oleic acid ranged from 65.36% in ‘Kotruvsi’ to 79.92% in ‘Unafka’, which also showed the highest oleic/linoleic ratio. All cultivar means in this study exceeded the 250 mg kg^−1^ screening reference for the European Food Safety Authority (EFSA)-related phenolic content, thereby showcasing the potential of Albanian monovarietal oils with respect to their health properties. Multivariate analyses provided complementary chemical and technological classification rather than a single quality ranking. These cultivar fingerprints provide a basis for conservation, breeding, authenticity assessment and future Protected Designation of Origin (PDO)/Protected Geographical Indication (PGI) valorisation.

## 1. Introduction

Olive (*Olea europaea* L.) is among the most emblematic perennial crops of the Mediterranean Basin, where it has shaped landscapes, diets and rural economies over millennia. Its principal product, virgin olive oil (VOO), is obtained solely by mechanical means and therefore retains a complex and nutritionally valuable minor fraction in its composition [1,2,3]. Alongside its central culinary role, virgin olive oil (VOO) is a defining component of the Mediterranean dietary pattern. Randomized clinical evidence supports the contribution of an EVOO-rich Mediterranean diet to cardiovascular prevention, although such benefits arise from the overall dietary pattern and should not be attributed to a single compound in isolation [4,5]. The value of VOO consequently rests on the convergence of nutritional composition, oxidative stability, sensory quality, authenticity and territorial identity [6,7,8,9].

The major lipid fraction of VOO is dominated by triacylglycerols rich in monounsaturated fatty acids, particularly oleic acid, while linoleic, palmitic and other fatty acids occur in cultivar-dependent proportions. The balance between oleic and polyunsaturated fatty acids is nutritionally relevant and strongly influences resistance to oxidation during storage. Large germplasm surveys have shown that fatty-acid profiles are subject to substantial genetic control, but also respond to crop season and climatic conditions, including temperature and rainfall during fruit development [10,11]. Thus, a high-oleic profile may be a useful selection trait, but it cannot be interpreted independently of genotype-by-environment responses or of the accompanying minor compounds.

Phenolic compounds are among the most important molecules of the VOO minor fraction in terms of nutritional and anti-oxidative properties [12]. Secoiridoid derivatives such as oleacein, oleocanthal, oleuropein aglycone and ligstroside aglycone, together with phenolic alcohols and flavones, contribute to antioxidant capacity, shelf life and the bitterness and pungency associated with fresh, high-quality oils [12]. Recent work has linked the stability of EVOO to oleacein and oleocanthal and has demonstrated how secoiridoid metabolism contributes to sensory perception [13,14]. These compounds are also relevant to the European health-claim framework for olive oil polyphenols [15]. Moreover, phenolic composition is modified by extraction conditions: malaxation time and atmosphere can alter individual secoiridoids and, in turn, oxidative stability [16,17,18]. Meaningful germplasm characterisation therefore requires controlled processing, compound-specific and integrated interpretation of most important molecules present in olive oil, rather than reliance on a single and isolated chemical parameter.

Cultivar or genetic factor is the major source of variation in both phenolic and fatty-acid profiles, but cultivar effect is influenced by a series of other factors, such as of environmental, crop management and technological process parameters, mostly in the case of phenolic compounds. Extensive surveys have demonstrated broad cultivar-associated diversity in VOO phenols and have shown that genetic and interannual factors can affect not only total abundance but also the relative distribution of individual cultivars [11,17,18,19]. More recent evidence indicates that fruit moisture, fatty-acid composition and cultivar-specific enzymatic activity jointly influence phenolic transfer and transformation during oil extraction [20]. Multi-environment and multiyear studies likewise show that growing site, harvest time and season can modify phenols, fatty acids and sensory attributes, with responses that differ among genotypes [21,22]. These findings make experimental standardization essential particularly for the olive germplasm characterisation [23].

Traditional local cultivars often possess unique combinations of environmental adaptability, high oil yield, and superior chemical quality that are missing in globally dominant cultivars. Characterizing these local cultivars is essential for supporting ex situ conservation, olive breeding, and product traceability. Furthermore, it drives the development of premium, niche products linked to specific geographic origins [8].

In Albania, olive cultivation has ancient roots and includes a rich diversity of cultivated olives and oleasters. Although Albanian olive germplasm has been described as a distinct genetic resource [24,25,26], Mediterranean olive diversity shows substantial evidence of admixture. Using 25 SSR loci, Díez et al. (2015) identified three broadly western, central and eastern Mediterranean gene pools together with numerous admixed (‘mosaic’) cultivars, indicating that gene flow played an important role in olive diversification [27]. A subsequent study based on 96 EST-SNPs recovered a similar three-group structure but classified 58% of 853 analysed accessions as admixed [28]. In that study, the Albanian cultivar ‘Kotruvsi’ was inferred as the offspring of the parental pair ‘Toffehi Tataouine’ and ‘Safrawi’, two founders associated with descendants distributed across multiple Mediterranean regions. These findings suggest that the distinctiveness of Albanian olive germplasm has evolved within a broader history of Mediterranean-wide genetic exchange.

Available studies have begun to characterize Albanian olive oils, although existing evidence remains disorganized across cultivars, production areas and analytical approaches. Phenolic profiling of oils from five Albanian cultivars revealed marked differences in phenolic composition and showed that both cultivar identity and geographical origin contributed to sample differentiation [29]. A subsequent review highlighted the production importance of olive cultivation in Albania and summarized evidence for cultivar- and region-dependent variation in fatty-acid composition, phenolic profiles and volatile compounds, while emphasizing the value of authenticity and geographical-origin certification for Albanian olive oils [30]. More recently, detailed characterisation of two commercial ‘Kalinjot’ EVOOs demonstrated variation in fatty acids, phenolics, volatiles and sensory quality, while short-term fruit-storage experiments with ‘Kalinjot’, ‘Leccino’ and ‘Frantoio’ highlighted the importance of pre-processing conditions [31]. Collectively, these studies establish the quality potential of Albanian oils, but they do not yet provide a unified comparison of a wider set of molecularly authenticated local cultivars grown and processed under the same conditions. A further limitation is that phenolic composition, fatty-acid composition and technological and agronomical traits are often evaluated separately. This can obscure multi-trait profiles in which high concentrations of bioactive secoiridoids coexist with favourable lipid stability and oil yield, or reveal trade-offs that are not apparent in univariate comparisons.

Against this background, the present study aimed to characterize the compositional and technological diversity of eleven authenticated olive cultivars maintained in the Albanian germplasm collection at Agricultural Technology Transfer Centre of Vlorë (QTTB Vlorë). The cultivars were evaluated in a common field and harvested at a standardised maturity stage during the 2025 harvest season, followed by uniform industrial-scale oil extraction. Specifically, the study sought to: (i) quantify ten individual phenolic compounds by LC-MS/MS and determine fatty-acid composition by GC-FID; (ii) compare fruit moisture and oil content together with the two chemical blocks; (iii) assess cultivar-associated variation using ANOVA analysis; and (iv) integrate phenolic, fatty-acid and technological information through principal component analysis and hierarchical clustering statistical analysis. We hypothesized that the authenticated Albanian cultivars would display distinct, complementary chemical profiles under common experimental conditions and that integrated analysis would identify multi-trait combinations of potential value for germplasm conservation, cultivar selection and the future valorisation of Albanian monovarietal oils.

## 2. Materials and Methods

### 2.1. Plant Material, Experimental Orchard and Sampling Design

A total of 11 Albanian olive cultivars (*Olea europaea* L.) maintained at the Germplasm Bank of the Agricultural Technology Transfer Centre of Vlorë (QTTB Vlorë), Albania, were evaluated: ‘Kalinjot’, ‘Karen’, ‘Koran’, ‘Kotruvsi’, ‘Managjel FV’, ‘Mixani’, ‘Pulazeqin’, ‘U.H. Himare’, ‘U.K. Berat’, ‘Ulliri i Kuq’ and ‘Unafka’. The cultivars are maintained in the ex situ olive field collection established in 1998–1999 at the QTTB Vlorë Experimental Production Base (40°35′35″ N, 19°28′54″ E). The autochthonous cultivars in the collection are represented by five homologous trees each, planted at a spacing of 6.0 × 6.5 m.

The trees were grown in the same experimental orchard under the common edaphoclimatic and crop-management conditions. The orchard is located on predominantly flat terrain with a gentle slope, and the soil is classified as a clay-loam. A drip-irrigation system is installed, although irrigation is applied only under conditions of severe water deficit because of the limited availability of irrigation water.

The area has a typical Mediterranean climate, characterised by mild, humid winters and hot, dry summers. The mean annual temperature ranges from 16 to 18 °C, with mean temperatures of 8–10 °C during the coldest months and 25–26 °C during the warmest months; minimum temperatures generally range from 0 to −2 °C. Mean annual precipitation is approximately 900–1000 mm.

Fruit was collected in October 2025 at a maturity index of 3, defined by reddish-to-violet skin pigmentation. Fruit ripening was tracked weekly starting in early October 2025 across all trees. For each tree, 100 fruits were randomly sampled around the canopy perimeter (at human height across all four cardinal directions) and classified into five visual colour categories (Classes 0–4) according to the harmonized methodology defined in the GEN4OLIVE project [23]. All cultivars were harvested in October 2025 upon reaching a standardised maturity index of 3.0 (characterised by reddish-to-violet skin pigmentation across the fruit population), minimising stage-dependent maturity bias during comparative compositional profiling.

For each of the 11 cultivars, five trees were independently sampled. The entire tree (approximately 100 kg) was manually harvested using a mechanical branch shaker. Olives from each tree were processed separately using an industrial mill to obtain a distinct oil batch. To explicitly capture intra-batch operational fluctuations during continuous industrial extraction, such as transient phase-separation dynamics and paste-conditioning noise within the decanter, two sequential oil samples were collected per tree batch at distinct time points (at the initiation and towards the completion of the oil outflow stream). This continuous-flow sampling strategy yielded 10 oil samples per cultivar (110 samples in total), capturing both biological tree-to-tree variability and operational processing noise across all evaluated cultivars under uniform mill operating parameters. All oil samples were subsequently filtered through cellulose filter paper, and a 100 mL aliquot of each was stored in amber glass bottles at −20 °C for three months until analysis. One month after extraction and storage, the samples were transported by air to the University of Córdoba (Spain) within 24 h under ambient conditions. Upon arrival, all oil samples were frozen again and analysed within two months. Prior to analytical processing, the frozen samples were thawed in the dark at room temperature (20–22 °C) for 24 h.

### 2.2. Cultivar Authentication by Simple Sequence Repeat Markers

The identity of the sampled trees was checked by the Varietal Identification Service of the Department of Agronomy, University of Cordoba (UCOLIVO research group, Spain). Genomic DNA extracted from young leaves was profiled using the simple sequence repeat (SSR) loci DCA3, DCA9, DCA11, DCA16, DCA18 and GAPU101. Multilocus profiles were compared with the World Olive Germplasm Bank of Córdoba University (WOGB-UCO) reference collection for true-to-type authentication and to resolve names, synonymies and homonymies [32]. The SSR data were not included in the chemical or chemometric analyses. The authenticated designations and relevant synonyms are reported in Supplementary Table S1.

### 2.3. Standardised Oil Extraction at a Commercial Mill

For each tree of each cultivar, approximately 50 kg of olives were processed as a separate batch at the Musai Olive Oil mill near QTTB Vlorë, Albania. Extraction was performed using a commercial three-phase continuous line installed by F.lli Ferri S.r.l. (Andria, Italy), with a nominal processing capacity of 2.5 t h^−1^. The processing line comprised a hammer crusher fitted with a rotating 6.5 mm grid, Green 7 series malaxers, an Alfa Laval X6 three-phase decanter and two Alfa Laval UVPX 510 vertical separators. To handle small individual batches without cross-contamination, the facility utilises six parallel malaxing units. Each ~100 kg batch was malaxed at 24 °C for 30 min in an isolated unit before being introduced sequentially into an Alfa Laval X6 three-phase decanter. Short intermediate water rinses were applied between consecutive cultivar batches to prevent residual carryover. Centrifugal phase separation for each batch was completed within approximately 1.5 min, during which two sequential oil stream samples were collected. Subsequently, the oil samples were decanted in laboratory 1 litter tubes for 6 h, and were then filtered using cellulose paper.

### 2.4. Fruit Moisture and Total Oil Content

Fruit technological parameters were measured at the Food Safety and Veterinary Institute (ISUV), Tirana, Albania. Moisture was determined gravimetrically on homogenised olive paste by drying a 10 g portion at 105 °C to constant mass. Total extractable oil was determined by Soxhlet extraction [33]. A dried portion was ground to a homogeneous particle size, transferred to a cellulose thimble and extracted continuously for 6–8 h with n-hexane (boiling point 69 °C). The solvent was subsequently removed, and the recovered lipid was dried and weighed to constant mass.

The Soxhlet result was expressed as oil content on a dry-matter basis (Oil_DM, %). Oil content on a fresh-matter basis (Oil_FW, %) was recalculated from the measured moisture and Oil_DM values as:

Oil_FW = Oil_DM × (100 − moisture)/100.

These variables reflect the total solvent-extractable oil content of the fruit rather than the yield or efficiency of the mechanical oil extraction process.

### 2.5. Phenolic Compound Analysis

Phenolic compounds were quantified at the Department of Analytical Chemistry, University of Cordoba, following the absolute liquid chromatography–tandem mass spectrometry (LC–MS/MS) procedure of Castillo-Luna and Priego-Capote (2026) [34]. Phenolic extracts were analysed using an UltiMate 3000 liquid chromatograph coupled to a TSQ Quantum Access MAX triple-quadrupole mass spectrometer equipped with a heated electrospray-ionisation source (Thermo Fisher Scientific, Dreieich, Germany). Detection was performed in negative-ion multiple-reaction-monitoring (MRM) mode. Compound-specific retention times, precursor and product ions, tube-lens voltages and collision energies are provided in Supplementary Table S2.

Matrix-matched calibration models were prepared in refined olive-pomace oil spiked with phenolic standards over 0.8–40 mg kg^−1^ (Supplementary Table S3). To maintain peak responses strictly within the established matrix-matched linear calibration range (0.8–40 mg kg^−1^), phenolic extracts were subjected to compound- and sample-specific serial dilutions (1:4, 1:10 and 1:40 *v*/*v*) prior to LC-MS/MS analysis. Absolute concentrations in the oil samples were subsequently calculated by applying the corresponding volumetric dilution factors to the interpolated standard curves for each compound. Analytical limits of detection (LOD, 0.05–0.25 mg kg^−1^) and quantification (LOQ, 0.15–0.80 mg kg^−1^) were determined based on signal-to-noise ratios of 3 and 10, respectively. Naringenin was used as the internal standard. Oleomissional and oleokoronal were quantified using the calibration responses of their corresponding closed monoaldehydic precursors, oleuropein aglycone and ligstroside aglycone, respectively. Concentrations were expressed in mg kg^−1^ oil for oleocanthal, oleacein, oleuropein aglycone, oleomissional, ligstroside aglycone, oleokoronal, hydroxytyrosol, luteolin, apigenin and tyrosol. Total quantified phenolics were calculated as the arithmetic sum of these 10 concentrations. It should be noted that non-targeted minor phenolic classes, such as lignans (+-pinoresinol and 1-acetoxypinoresinol) and phenolic acids, were not monitored in this MRM panel; this sum therefore represents a conservative, compound-specific metric rather than the exhaustive polar phenolic pool.

To avoid ambiguity among alternative secoiridoid nomenclatures, the following equivalences were used in this study. 3,4-DHPEA-EDA denotes the dialdehydic decarboxymethyl form of oleuropein aglycone, commonly known as oleacein; p-HPEA-EDA denotes the dialdehydic decarboxymethyl form of ligstroside aglycone, commonly known as oleocanthal. 3,4-DHPEA-EA denotes the closed-ring and open-ring aldehydic isomers of oleuropein aglycone, reported here as oleuropein aglycone and oleomissional, corresponding to closed-ring and open-ring isomers respectively; p-HPEA-EA denotes the corresponding closed-ring and open-ring aldehydic isomers of ligstroside aglycone, reported here collectively as ligstroside aglycone and oleokoronal, corresponding to closed-ring and open-ring isomers respectively. Accordingly, oleacein and oleomissional were interpreted as members of the oleuropein/hydroxytyrosol-related family, whereas oleocanthal and oleokoronal were interpreted as members of the ligstroside/tyrosol-related family [34].

For a screening index based on the European Food Safety Authority (EFSA), the measured mass concentrations of hydroxytyrosol, tyrosol, oleocanthal, oleacein, oleuropein aglycone, oleomissional, ligstroside aglycone and oleokoronal were summed. The resulting value was compared descriptively with 250 mg kg^−1^, equivalent to 5 mg per 20 g oil in Commission Regulation (EU) No 432/2012 [35]. This direct mass-based comparison was treated as a screening reference and not as independent legal certification of eligibility to bear the health claim.

### 2.6. Fatty-Acid Composition Analysis

Fatty-acid methyl esters (FAMEs) composition was determined at the Department of Analytical Chemistry, University of Cordoba. They were prepared by cold transesterification according to a modified International Olive Council (IOC) method (COI/T.20/Doc. No 33/Rev.1; [36]). Transesterification was performed by vortexing a 0.05 g aliquot of olive oil with 1 mL of 0.5 N methanolic potassium hydroxide at 30 °C for 10 min. To induce phase separation, 1 mL of *n*-hexane was introduced and shaken for 5 min. The upper hexane layer containing fatty-acid methyl esters (FAMEs) was collected and diluted 1:25 (*v*/*v*) in n-hexane prior to injecting a 1 µL aliquot into the GC–FID system.

FAME separation was executed on an Agilent 7820A gas chromatograph equipped with an autosampler, a split/splitless injector, and an FID (Agilent Technologies, Santa Clara, CA, USA). Chromatographic resolution was achieved using an SP™-2560 fused-silica capillary column (100 m × 0.25 mm I.D., 0.2 µm film thickness; Supelco, Bellefonte, PA, USA). Instrumental conditions were set as follows: injector temperature at 250 °C, splitless injection mode, and carrier gas flow rate at 1.0 mL min^−1^. The oven thermal gradient was programmed as follows: initial temperature of 100 °C (held for 5 min), increased at 4 °C min^−1^ to 240 °C (held for 20 min), and finally ramped at 10 °C min^−1^ to 250 °C (held for 4 min), giving a total chromatographic run time of 65 min, supplemented by a 10 min post-run period for system re-equilibration. Peak identification was achieved by comparing individual retention times against those of a commercial Supelco 37-component FAME standard mixture (Sigma-Aldrich. St. Louis, MO, USA).

### 2.7. Data Processing and Univariate Statistics

The complete matrix contained 110 observations, with 10 oil samples per cultivar. Cultivar effects were evaluated separately for the phenolic compounds, fatty acids and technological parameters (moisture and oil content in fresh and dry matter). For each response, the identity, log(1 + x), square-root, Yeo–Johnson and, for positive values, Box–Cox transformations were evaluated (Supplementary Table S4). The transformation providing the best combined residual normality, and variance homogeneity was retained. Residual normality was assessed using the Shapiro–Wilk test, and homogeneity of variance was assessed using the median-centred Levene test.

Cultivars were compared by one-way analysis of variance (ANOVA), followed by Tukey’s honestly significant difference (HSD) test at *p* < 0.05. Effect size was expressed as η^2^. Tukey-adjusted pairwise comparisons were converted to compact-letter displays; means sharing at least one letter were not significantly different. Means and standard deviations were reported on the original measurement scale, independent of the transformation used for statistical testing.

Univariate analyses were performed in Python 3.12.13. Data management used pandas 3.0.5 and NumPy 2.5.1. Transformations, the Shapiro–Wilk test, the Levene test and one-way ANOVA were implemented with SciPy 1.18.0. Tukey’s HSD test was implemented with statsmodels 0.14.6, and compact-letter displays were constructed with NetworkX 3.6.1.

### 2.8. Hierarchical Clustering, Principal Component Analysis and Correlations

Hierarchical cluster analysis (HCA) was performed first to identify groups of cultivars with similar chemical profiles. Separate analyses were conducted for the 10 individual phenolic compounds, the 13 fatty acids and the joint matrix containing the 10 phenolics, 13 fatty acids and 3 technological parameters. Before analysis, every variable was converted to a standard score (z-score). HCA was applied to the z-scored cultivar means using Euclidean distance and Ward’s linkage. The phenolic, fatty-acid and joint analyses retained 4, 2 and 3 exploratory chemometric clusters, respectively. Cluster consistency was examined using mean silhouette width and 1000 within-cultivar bootstrap resamples.

Principal component analysis (PCA) was subsequently applied to the same z-scored samples matrices to visualise the HCA groups in a reduced-dimensional space. The HCA cluster assigned to each cultivar was transferred to the PCA score plot as the point colour. Cultivars were distinguished by symbols, stars represented cluster centroids and shaded regions represented 95% covariance ellipses. Loading vectors indicated the variables contributing most strongly to the first 2 principal components (PC1 and PC2). Total quantified phenolics and derived ratios were excluded from the active PCA matrices because they were calculated from variables already included in the models.

Pearson correlation coefficients were calculated as exploratory descriptions of pairwise relationships among the 10 phenolic compounds, among the 13 fatty-acid percentages and between phenolic and fatty-acid variables. Benjamini–Hochberg false-discovery-rate correction was applied separately within each correlation family, and only associations with q < 0.05 were displayed. Z-score standardisation and PCA were implemented with scikit-learn 1.9.0; HCA and Pearson correlations were implemented with SciPy 1.18.0. Figures were produced using Matplotlib 3.11.1 and seaborn 0.13.2.

## 3. Results

Based on the analytical results of the olive samples belonging to the 11 cultivars, one-way ANOVA showed a significant cultivar effect for every quantified phenolic compound, fatty acid, derived ratio and technological parameter—moisture and fruit oil content (*p* < 0.001; Supplementary Table S4). The proportion of variance attributable to cultivar was high for the phenolic responses (η^2^ = 0.574–0.999) and fatty-acid responses (η^2^ = 0.972–0.998), and moderate to high for the technological parameters (η^2^ = 0.412–0.621). The lowest effect sizes were obtained for dry-matter oil content (η^2^ = 0.412) and fruit moisture (η^2^ = 0.551). Tukey groupings are presented on figures and tables together with means and standard deviations on the original measurement scale.

### 3.1. Cultivar Differentiation on Phenolic Compounds

**Figure 1:**
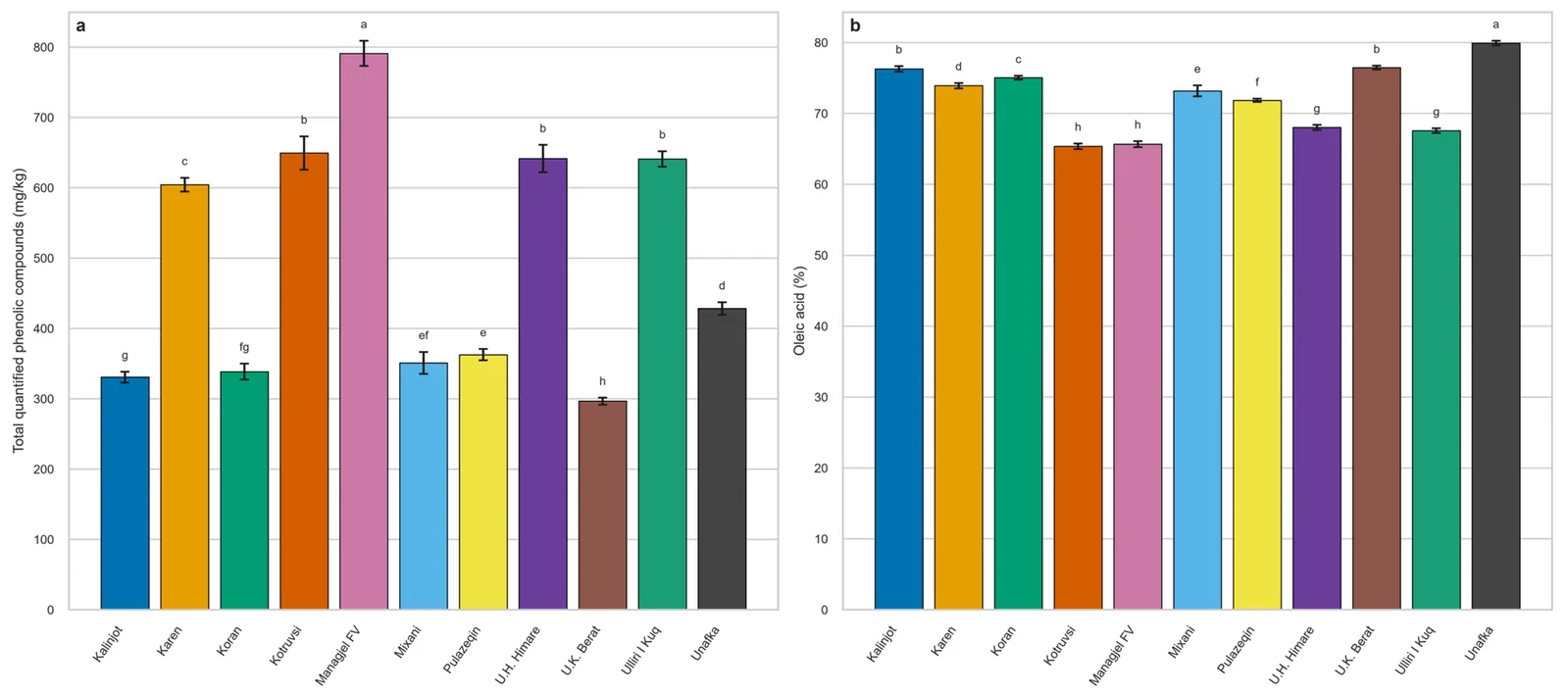
Cultivar differences in (**a**) total quantified phenolic compounds and (**b**) oleic acid. Bars show means, and error bars show standard deviations. Within each panel, means with different lowercase letters differed significantly (*p* < 0.05).

Total quantified phenolics differed markedly among cultivars (Figure 1a; Table 1 and Table 2). Mean concentrations ranged from 296.5 ± 5.0 mg kg^−1^ in ‘U.K. Berat’ to 791.0 ± 17.9 mg kg^−1^ in ‘Managjel FV’. ‘Managjel FV’ formed the highest Tukey group, whereas ‘Kotruvsi’, ‘U.H. Himare’ and ‘UlliIi i Kuq’ formed a second high group with similar means of 649.3, 641.4 and 640.9 mg kg^−1^, respectively. ‘Karen’ also showed a comparatively high concentration (604.3 ± 9.8 mg kg^−1^). At the lower end, ‘Kalinjot’, ‘Koran’, ‘Mixani’ and ‘Pulazeqin’ contained approximately 331–363 mg kg^−1^, while ‘U.K. Berat’ had the lowest total.

**Figure 2:**
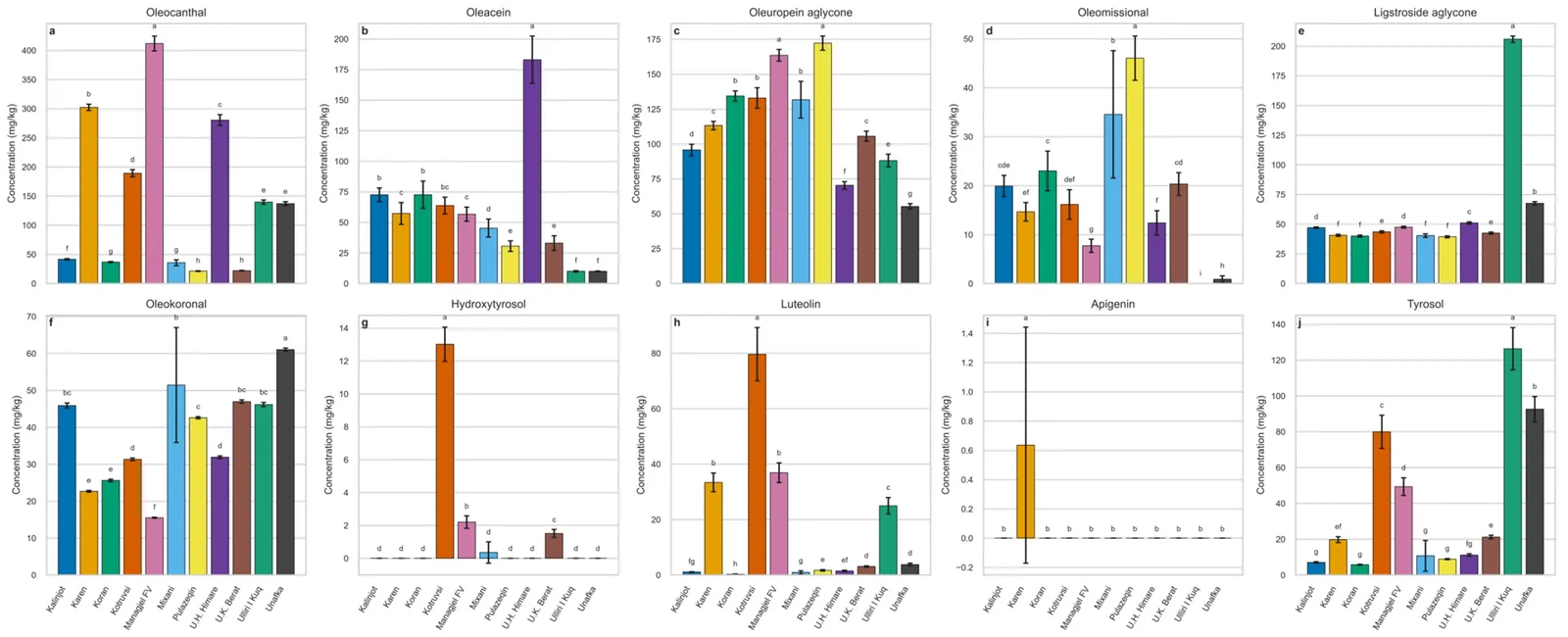
Absolute concentrations of the 10 quantified phenolic compounds in oils from the 11 olive cultivars: (**a**) oleocanthal, (**b**) oleacein, (**c**) oleuropein aglycone, (**d**) oleomissional, (**e**) ligstroside aglycone, (**f**) oleokoronal, (**g**) hydroxytyrosol, (**h**) luteolin, (**i**) apigenin and (**j**) tyrosol. Bars show means, and error bars show standard deviations. Within each panel, means sharing at least one lowercase letter were not significantly different (*p* < 0.05).

The cultivar differences in total concentration resulted from distinct compound-specific profiles (Figure 2; Supplementary Figure S1; Table 1). Oleocanthal was highest in ‘Managjel FV’ (411.7 ± 12.8 mg kg^−1^), followed by ‘Karen’ (302.3 ± 5.4 mg kg^−1^) and ‘U.H. Himare’ (280.3 ± 9.3 mg kg^−1^). Oleacein followed a different pattern and reached its maximum in ‘U.H. Himare’ (183.0 ± 19.4 mg kg^−1^). ‘Pulazeqin’ had the highest oleuropein aglycone and oleomissional concentrations (172.3 ± 5.1 and 46.0 ± 4.5 mg kg^−1^, respectively), whereas ‘Ulliri i Kuq’ was distinguished by ligstroside aglycone (205.8 ± 2.7 mg kg^−1^) and tyrosol (126.3 ± 11.8 mg kg^−1^). ‘Kotruvsi’ contained the highest luteolin concentration (79.6 ± 9.6 mg kg^−1^) and was the only cultivar with a mean hydroxytyrosol concentration above 10 mg kg^−1^ (13.0 ± 1.0 mg kg^−1^). Apigenin was negligible in most oils and was measurable principally in ‘Karen’. Thus, cultivars with similar totals could still have clearly different phenolic fingerprints.

The relative phenolic concentration, expressed as percentages of each cultivar’s total quantified phenolics, also showed important differences in compound dominance among cultivars (Supplementary Figure S1). Oleocanthal represented approximately half of the profile in ‘Karen’ (50.0%) and ‘Managjel FV’ (52.1%), while ‘U.H. Himare’ was co-dominated by oleocanthal (43.7%) and oleacein (28.5%). Oleuropein aglycone was the largest fraction in ‘Koran’ (39.7%), ‘Mixani’ (37.7%), ‘Pulazeqin’ (47.5%) and ‘U.K. Berat’ (35.6%). ‘Ulliri i Kuq’ showed the largest ligstroside-aglycone fraction (32.1%) together with tyrosol (19.7%), whereas ‘Unafka’ combined oleocanthal (32.0%) with tyrosol (21.6%). ‘Kotruvsi’ had a more distributed profile, in which oleocanthal (29.1%) and oleuropein aglycone (20.5%) were accompanied by similarly large luteolin and tyrosol fractions (12.3% each). This phenolic relative view therefore clarified which compounds dominated each cultivar fingerprint.

The exploratory Pearson correlation matrix showed coordinated variation among several phenolic compounds (Supplementary Figure S2). Hydroxytyrosol was strongly associated with luteolin (r = 0.843), and ligstroside aglycone was associated with tyrosol (r = 0.749); the latter within-family relationship is consistent with their shared ligstroside/tyrosol-related classification. Oleocanthal (p-HPEA-EDA) was inversely related to oleokoronal (p-HPEA-EA; r = −0.633), indicating that cultivars rich in the dialdehydic decarboxymethyl form tended not to be rich in the aldehydic EA isomers of the same ligstroside-derived family. Oleomissional (3,4-DHPEA-EA) was inversely related to tyrosol (r = −0.645), an across-family contrast between oleuropein/hydroxytyrosol- and ligstroside/tyrosol-related compounds. Because these are cultivar-structured observational correlations, they describe co-variation rather than direct precursor–product conversion.

### 3.2. Cultivar Clustering Based on Phenolic Composition

**Figure 3:**
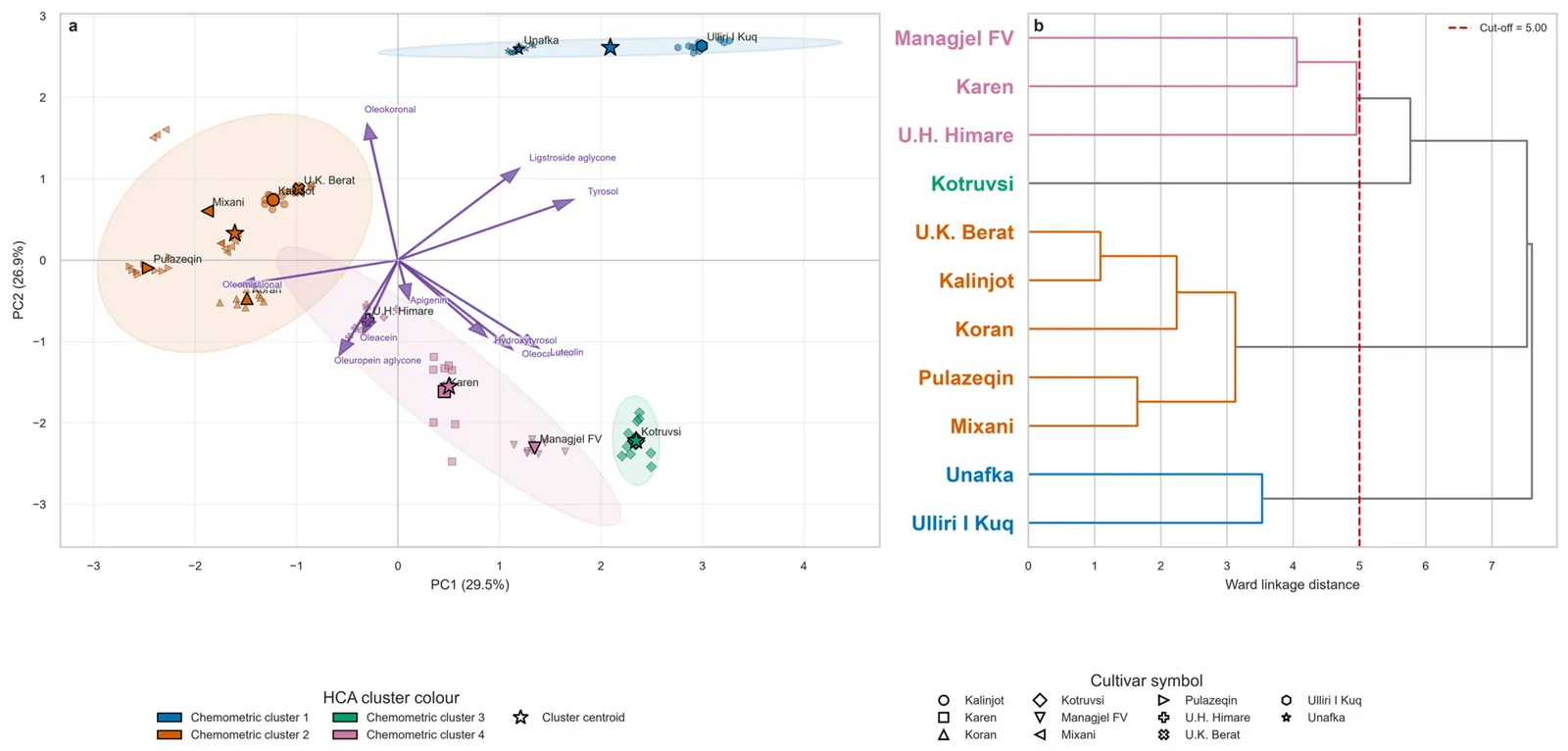
Phenolic chemometric cultivar clustering. (**a**) Principal component analysis score-loading biplot based on the 10 z-standardised individual phenolics. Points represent independent sample-level oils; point symbols identify cultivars, and colours identify the four hierarchical cluster analysis groups. Large cultivar symbols mark cultivar centroids, stars mark cluster centroids and shaded regions are 95% covariance ellipses. (**b**) Ward-linkage dendrogram of z-standardised cultivar means. The red dashed line is the Ward distance cut-off used to define four clusters; branch colours match the cluster colours in panel (**a**).

HCA separated the cultivars into four exploratory phenolic clusters (Figure 3b; Supplementary Table S5). Cluster 1 comprised ‘Ulliri i Kuq’ and ‘Unafka’; cluster 2 comprised ‘Kalinjot’, ‘Koran’, ‘Mixani’, ‘Pulazeqin’ and ‘U.K. Berat’; cluster 3 contained only ‘Kotruvsi’; and cluster 4 comprised ‘Karen’, ‘Managjel FV’ and ‘U.H. Himare’. Mean pairwise bootstrap co-membership was 100.0% for clusters 1 and 2 and 99.9% for cluster 4 (Supplementary Table S6).

PCA provided a 2-dimensional representation of the HCA groups (Figure 3a). PC1 and PC2 explained 29.5% and 26.9% of the z-scored phenolic variance, respectively, giving a cumulative value of 56.4%. Tyrosol, luteolin and ligstroside aglycone contributed positively to PC1, whereas oleomissional contributed in the opposite direction. PC2 was associated positively with oleokoronal and ligstroside aglycone and negatively with oleuropein aglycone, oleocanthal, luteolin and oleacein. Thus, the principal separation did not reduce to oleuropein versus ligstroside origin; PC2 also opposed molecular forms within the ligstroside-related family, with oleokoronal and ligstroside aglycone loading opposite oleocanthal, while oleacein and oleuropein aglycone shared negative PC2 loadings. The positions of ‘Ulliri i Kuq’, ‘Kotruvsi’ and the oleocanthal-rich cultivars were consistent with their distinctive individual phenolic profiles. All 10 loading vectors are displayed in the biplot, and their numerical loadings and contributions to PC1 and PC2 are reported in Supplementary Table S7A.

### 3.3. Fatty-Acid Composition of Evaluated Cultivars

Oleic acid was the predominant fatty acid in every cultivar but varied by 14.6 percentage points (Figure 1b; Table 2 and Table 3; Supplementary Figure S3). ‘Unafka’ had the highest concentration (79.92 ± 0.33%) and formed a separate Tukey group. ‘Kalinjot’ and ‘U.K. Berat’ followed with values above 76%, whereas ‘Kotruvsi’ and ‘Managjel FV’ had the lowest means (65.36 ± 0.39% and 65.65 ± 0.42%, respectively). Linoleic acid showed the opposite broad pattern, ranging from 4.88 ± 0.16% in ‘Unafka’ to 16.80 ± 0.28% in ‘Kotruvsi’. Consequently, the oleic-to-linoleic acid ratio ranged from 3.89 ± 0.08 in ‘Kotruvsi’ to 16.39 ± 0.59 in ‘Unafka’. Palmitic acid was lowest in ‘Kalinjot’ and highest in ‘Managjel FV’.

**Figure 4:**
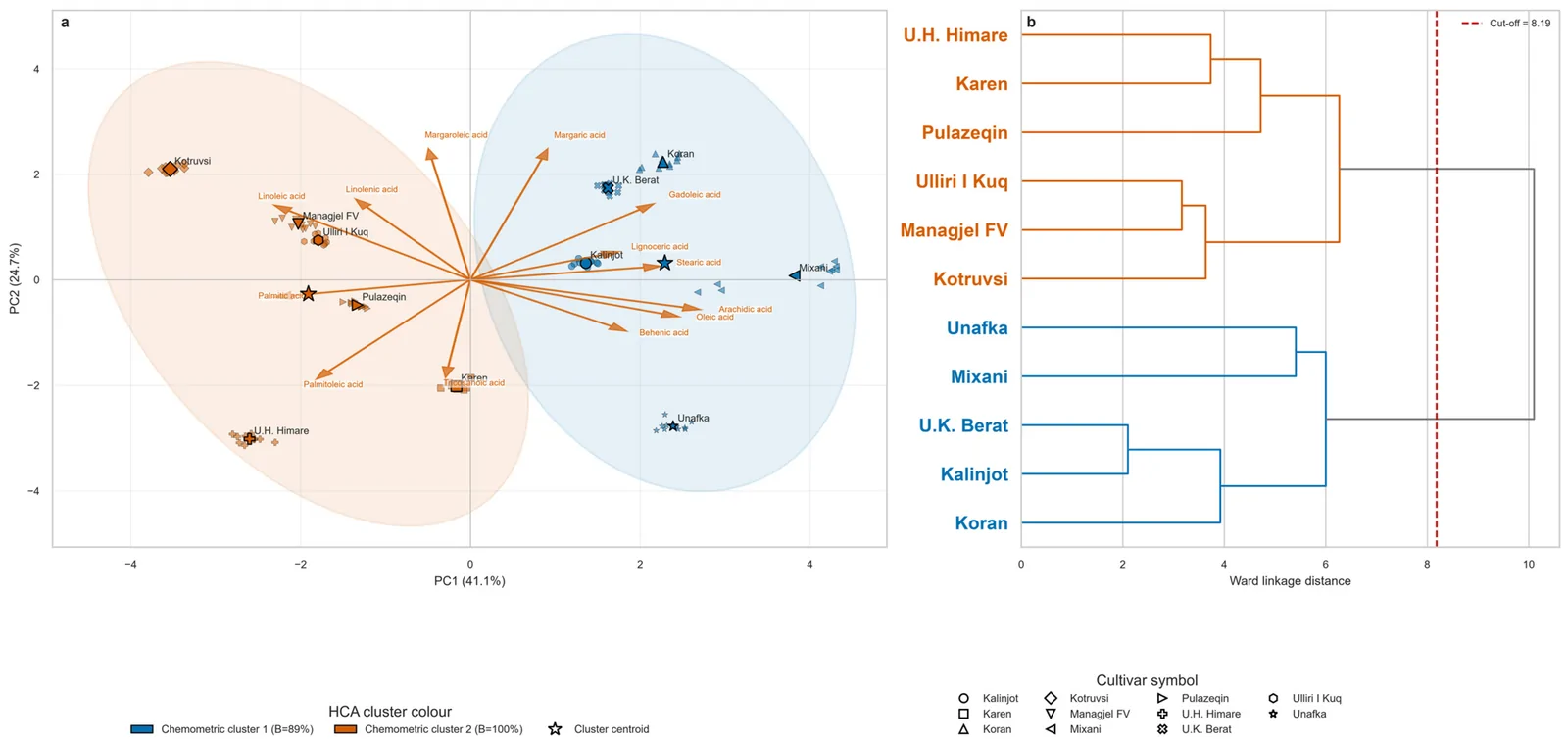
Fatty-acid chemometric cultivar clustering. (**a**) Principal component analysis score-loading biplot based on 13 z-standardised fatty-acid percentages. Points represent independent sample-level oils; point symbols identify cultivars, and colours identify the two hierarchical cluster analysis groups. Large cultivar symbols mark cultivar centroids, stars mark cluster centroids and shaded regions are 95% covariance ellipses. (**b**) Ward-linkage dendrogram of z-standardised cultivar means; the red dashed line shows the cut-off defining two clusters. Branch colours match panel (**a**).

HCA of the z-scored fatty-acid profiles retained two clusters (Figure 4b; Supplementary Table S5). Cluster 1, which was richer in oleic acid content, contained ‘Kalinjot’, ‘Koran’, ‘Mixani’, ‘U.K. Berat’ and ‘Unafka’, while cluster 2 contained ‘Karen’, ‘Kotruvsi’, ‘Managjel FV’, ‘Pulazeqin’, ‘U.H. Himare’ and ‘Ulliri i Kuq’. Bootstrap co-membership was 88.6% and 100.0% for clusters 1 and 2, respectively (Supplementary Table S6).

In the PCA representation, PC1 and PC2 explained 41.1% and 24.7% of the z-scored fatty-acid variance, respectively (65.9% cumulatively; Figure 4a). Oleic, arachidic, stearic and gadoleic acids contributed positively to PC1, opposed mainly by linoleic and palmitic acids. PC2 was influenced positively by margaric and margaroleic acids and negatively by palmitoleic and tricosanoic acids. All 13 fatty-acid loading vectors are shown, with numerical loadings and PC1–PC2 contributions in Supplementary Table S7B. Exploratory cross-block correlations reflected the same broad gradients: total quantified phenolics correlated positively with palmitic acid (r = 0.864) and negatively with oleic acid (r = −0.790), while luteolin correlated positively with linoleic acid (r = 0.749; Supplementary Figure S5).

The exploratory fatty-acid correlation matrix was dominated by an inverse oleic–linoleic relationship (r = −0.926) and an inverse palmitic–oleic relationship (r = −0.896; Supplementary Figure S8). Positive correlation connected stearic with arachidic acid (r = 0.816), arachidic with behenic acid (r = 0.799) and behenic with lignoceric acid (r = 0.728), a pattern compatible with coordinated abundance along saturated-chain elongation. Margaric and margaroleic acids were also positively correlated (r = 0.842). These correlation patterns suggest cultivar-specific differences in fatty-acids desaturation and elongation.

### 3.4. Health-Related Compounds and Technological Parameters

**Figure 5:**
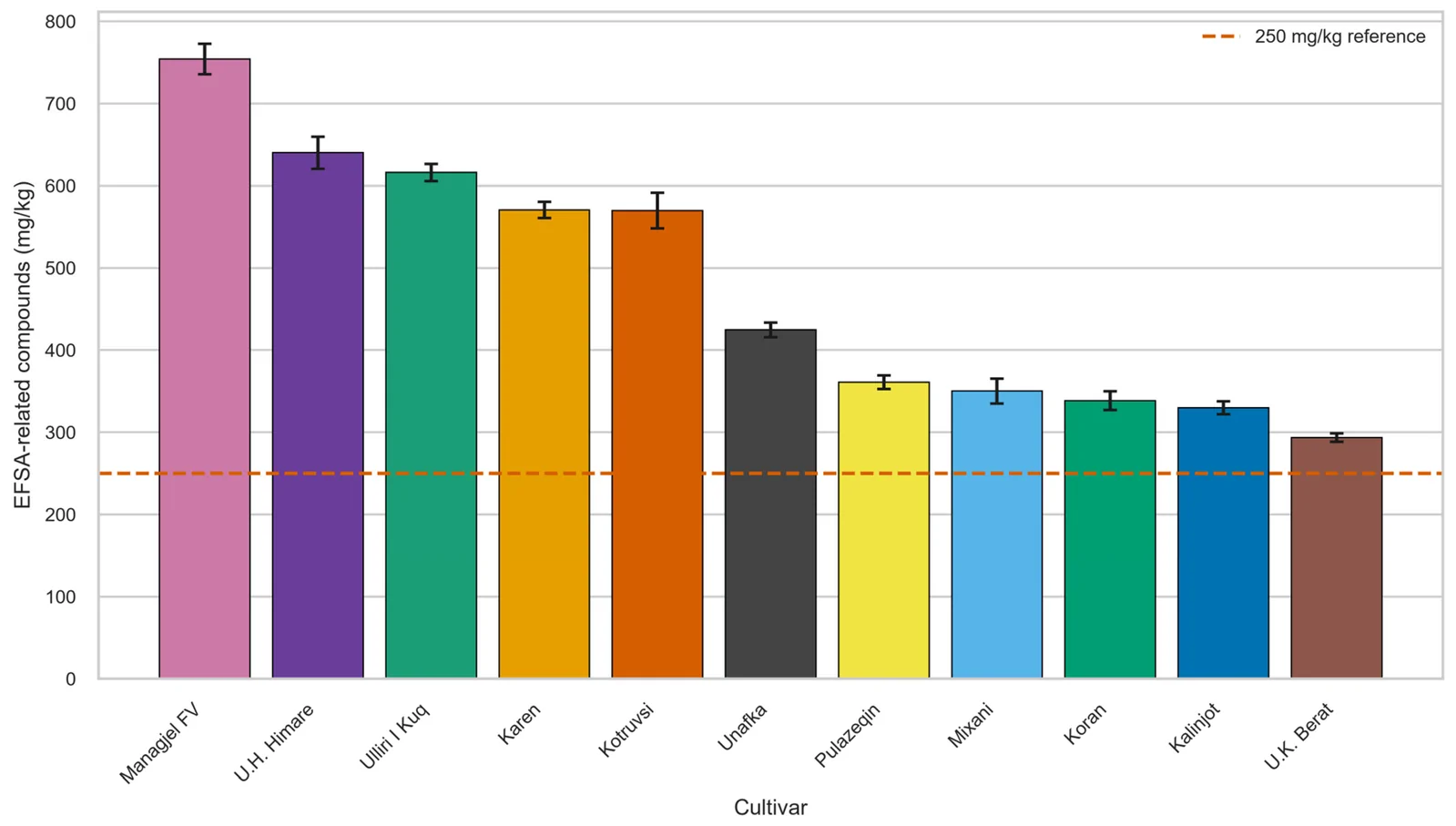
Direct mass sum of hydroxytyrosol, tyrosol, oleocanthal, oleacein, oleuropein aglycone, oleomissional, ligstroside aglycone and oleokoronal in oils from the 11 cultivars. Bars show means, and error bars show standard deviations. The orange dashed line indicates 250 mg kg^−1^, equivalent by mass to 5 mg per 20 g oil. Abbreviation: EFSA, European Food Safety Authority.

The direct mass sum of hydroxytyrosol, tyrosol and the six quantified secoiridoid derivatives ranged from 293.5 ± 5.1 mg kg^−1^ in ‘U.K. Berat’ to 754.1 ± 18.6 mg kg^−1^ in ‘Managjel FV’ (Figure 5; Table 2). Every cultivar mean exceeded the descriptive reference of 250 mg kg^−1^ corresponding to 5 mg per 20 g oil. ‘U.H. Himare’ and ‘Ulliri i Kuq’ also exceeded 600 mg kg^−1^. These values indicate high concentrations of compounds related to the health claim, while remaining a mass-based screening comparison rather than formal regulatory certification.

The bivariate screening of the EFSA-related sum and the oleic-to-linoleic acid ratio showed that the two traits did not vary in parallel (Supplementary Figure S4). ‘Karen’ was above the collection median for both variables. ‘Managjel FV’, ‘U.H. Himare’, ‘Ulliri i Kuq’ and ‘Kotruvsi’ combined high EFSA-related sums with below-median oleic-to-linoleic ratios, whereas ‘Unafka’ combined the highest ratio with an intermediate phenolic-derivative sum.

Regarding the technological parameters, fruit moisture ranged from 45.6 ± 1.9% in ‘U.H. Himare’ to 53.3 ± 2.3% in ‘U.K. Berat’ (Supplementary Figure S6; Table 2). Fresh-matter oil content ranged from 22.2 ± 2.2% in ‘U.K. Berat’ to 29.9 ± 2.0% in ‘U.H. Himare’. ‘Ulliri i Kuq’, ‘Kalinjot’ and ‘Karen’ also showed comparatively high fresh-matter oil contents. Dry-matter oil content ranged from 47.5 ± 4.3% in ‘U.K. Berat’ to 55.0 ± 2.5% in ‘U.H. Himare’, with a similar value in ‘Ulliri Kuq’ (54.7 ± 4.1%).

### 3.5. Joint Chemical and Technological Differentiation of Evaluated Cultivars

HCA of the joint z-scored matrix retained three exploratory chemometric clusters (Supplementary Figure S7b; Supplementary Table S5). Cluster 1 included ‘Kalinjot’, ‘Koran’, ‘Mixani’, ‘Pulazeqin’, ‘U.K. Berat’ and ‘Unafka’; cluster 2 included ‘Karen’ and ‘U.H. Himare’; and cluster 3 included ‘Kotruvsi’, ‘Managjel FV’ and ‘Ulliri i Kuq’. Bootstrap co-membership was 95.0%, 99.8% and 86.8% for clusters 1, 2 and 3, respectively (Supplementary Table S6).

The subsequent PCA representation explained 30.1% of the joint z-scored variance on PC1 and 18.2% on PC2 (48.3% cumulatively; Supplementary Figure S7a; Supplementary Table S7C). PC1 contrasted oleic, arachidic and stearic acids with oleocanthal, luteolin, linoleic and palmitic acids and the oil-content variables; PC2 contrasted oleuropein aglycone, hydroxytyrosol and luteolin with oleacein, oleocanthal and dry-matter oil content. Accordingly, cluster 1 occupied mainly positive PC1 and combined the highest mean oleic acid (75.45%) with the lowest total phenolics, health-claim-related phenolic sum and dry-matter oil content (351.2 mg kg^−1^, 349.4 mg kg^−1^ and 49.80%); ‘Pulazeqin’ was an exception because of its high oleuropein aglycone. Cluster 2 combined high total phenolics (622.9 mg kg^−1^), high oleocanthal and oleacein (291.3 and 120.1 mg kg^−1^), intermediate oleic acid (70.96%) and the highest dry-matter oil content (53.97%). Under the nomenclature defined in the Methods section, this cluster was relatively EDA-rich across both the ligstroside-derived and oleuropein-derived families. Cluster 3 had the highest total and health-claim-related phenolics (693.7 and 646.6 mg kg^−1^), the highest mean oleuropein and ligstroside aglycones (128.2 and 98.9 mg kg^−1^), the lowest oleic acid (66.19%) and intermediate-high dry-matter oil content (51.70%); it was therefore relatively aglycone-rich across both families. These descriptors summarize the dominant compound forms and do not establish pathway flux. Thus, the clusters capture complementary phenolic, oleic-acid and oil-accumulation profiles rather than a single quality ranking. All 26 loading vectors are displayed and quantified in Supplementary Table S7C.

## 4. Discussion

### 4.1. Cultivar-Associated Phenolic Diversification

The present study reveals marked and trait-specific compositional differentiation among the eleven Albanian olive cultivars. Cultivar effects were significant for every measured response, explaining 57.4–99.9% of phenolic variance, 97.2–99.8% of fatty-acid variance and 41.2–62.1% of technological variance. Oil samples of those cultivars were obtained from one single field, harvested at a comparable maturity stage and processed with one protocol, reducing in this way several environmental and technological sources of heterogeneity that complicate comparisons among commercial oils. The results therefore provide strong evidence of cultivar-associated differentiation under the conditions tested. They should not, however, be interpreted as unchangeable genetic values, because crop season, site and genotype-by-environment interactions can materially alter mostly phenols but also the fatty-acid profiles [17,18,19,37]. A previous work conducted by Kyçyk et al. (2021) [38] characterised several Albanian cultivars under common environmental conditions in the Córdoba Germplasm Bank (Spain), demonstrating cultivar-dependent differences in fatty-acid composition, total phenolics, tocopherols and oxidative stability. The present study extends these findings by evaluating genetically authenticated Albanian cultivars under their native production conditions and by applying targeted LC-MS/MS targeted phenolic profiling together with chemometric analyses. Although direct comparison of total phenolic contents is not appropriate because of the different analytical methodologies employed, both studies consistently identify cultivars such as ‘Ulliri i Kuq’, ‘Kalinjot’ and ‘Kotruvsi’ as characterised by favourable compositional traits, reinforcing their potential for premium monovarietal olive oil production [38].

Total quantified phenolics varied 2.7-fold, from 296.5 mg/kg in ‘U.K. Berat’ to 791.0 mg/kg in ‘Managjel FV’. The upper values for ‘Managjel FV’, ‘Kotruvsi’, ‘U.H. Himare’ and ‘Ulliri i Kuq’ demonstrate that Albanian germplasm includes several phenol-rich resources rather than a single outstanding cultivar. Their concentrations exceed those reported for the five Albanian oils examined by LC-DAD-ESI-MS/MS by Topi et al. (2020) [29], but the comparison is not direct because the earlier study involved different cultivars, regions, maturity indices, extraction conditions and analytical response models. They are also compatible with the broad Mediterranean variability documented in extensive field and harvest-period surveys, in which secoiridoid-rich cultivars can retain several hundred milligrams per kilogram while other cultivars decline sharply during ripening [39,40]. Accordingly, the main contribution here is not a claim of globally unprecedented concentrations, but the controlled identification of high-value phenotypes within an under-characterised national collection. When contextualised within broader international oil quality, the total phenolic levels of top Albanian cultivars align closely with established international benchmark cultivars. For instance, total phenolic concentrations in ‘Managjel FV’ (791.0 mg kg), ‘Kotruvsi’ (649.3 mg kg), and ‘U.H. Himare’ (641.4 mg kg) are comparable to, or even surpass, those reported for high-phenolic Mediterranean cultivars such as ‘Picual’ (500–800 mg/kg) and ‘Koroneiki’ (400–750 mg/kg) under controlled collection conditions. In particular, the oleocanthal concentration in ‘Managjel FV’ (411.7 mg kg) represents an exceptionally high value, exceeding typical averages for ‘Picual’ and ‘Cornicabra’. Conversely, cultivars at the lower end of the Albanian collection, such as ‘U.K. Berat’ (296.5 mg/kg) and ‘Kalinjot’ (330.7 mg/kg), exhibit phenolic loads comparable to moderate-producing cultivars like ‘Arbequina’ and ‘Arbosana’ (200–350 mg/kg) [19,41,42].

The individual profiles are more informative than total phenolics alone. ‘Managjel FV’ was characterised by the highest oleocanthal concentration, ‘U.H. Himare’ by the combination of oleocanthal and oleacein, ‘Pulazeqin’ by oleuropein aglycone and oleomissional, ‘Ulliri i Kuq’ by ligstroside aglycone and tyrosol, and ‘Kotruvsi’ by luteolin and hydroxytyrosol. These profiles span both precursor-related families and several molecular forms: oleacein (3,4-DHPEA-EDA) and oleomissional (3,4-DHPEA-EA) were assigned to the oleuropein/hydroxytyrosol-related family, whereas oleocanthal (p-HPEA-EDA), oleokoronal (p-HPEA-EA), ligstroside aglycone and tyrosol were assigned to the ligstroside/tyrosol-related family. This compositional determination is relevant because oils with similar total concentrations may differ in sensory properties, oxidative stability and the balance of bioactive secoiridoids [14,16,43].

These contrasts are biologically plausible in view of cultivar-dependent regulation of secoiridoid metabolism. Olive β-glucosidase plays a key role in the hydrolysis of phenolic glycosides during olive fruit processing. The OeGLU gene encodes a defence-related β-glucosidase that deglycosylates oleuropein with strong substrate specificity, converting it into a potent protein cross-linking agent [44]. Another β-glucosidase gene, OepGLU, isolated from cultivar Picual and recombinantly expressed, encodes an enzyme that displays the highest activity toward oleuropein, followed by ligstroside (25.6% relative activity) and demethyloleuropein (15.6%), generating the corresponding aglycone isomers in each case [20]. Olive β-glucosidase gene expression is developmentally regulated and cultivar-dependent and is also modulated by environmental factors including water regime, temperature, and light [20,44,45]. Subsequent chemical and enzymatic transformations during crushing and malaxation further shape the phenolic profile of the resulting oil.

The positive ligstroside aglycone–tyrosol correlation is therefore compatible with their common ligstroside/tyrosol-related family, whereas the negative oleocanthal–oleokoronal correlation may reflect cultivar-level partitioning between EDA and EA forms within that family. The negative oleomissional–tyrosol correlation instead represents an across-family contrast. Nevertheless, common precursor molecules, enzyme compartmentalisation, coordinated cultivar regulation and processing kinetics can all produce indirect associations between products, including inverse associations that do not correspond to a single reaction. Enzyme activity and pathway flux were not measured in this study, and repeated oils from the same cultivar share much of their biochemical background; the observed correlations should therefore be treated as pathway-informed hypotheses, not demonstrated precursor–product conversions.

### 4.2. Potential Implications of the Phenolic Profiles in Oil Sensory, Stability and Health-Claim Attributes

The compound-specific differences generate oil organoleptic property hypotheses. Specifically, elevated concentrations of dialdehydic decarboxymethyl secoiridoid derivatives, namely oleocanthal (*p*-HPEA-EDA) in ‘Managjel FV’ (411.7 mg/kg) and ‘Karen’ (302.3 mg/kg), as well as the co-dominance of oleacein (3,4-DHPEA-EDA) and oleocanthal in ‘U.H. Himare’, are anticipated to impart intense, persistent pungency sensation [7]. Conversely, cultivars rich in aglycone isomers and monoaldehydic forms, such as ‘Pulazeqin’ (characterised by high oleuropein aglycone, 172.3 mg/kg) and ‘Ulliri i Kuq’ (rich in ligstroside aglycone, 205.8 mg/kg), are expected to exhibit a predominantly bitter sensory profile [46]. Although official sensory panel evaluation remains to be conducted in prospective multi-year trials, these distinctive chemical fingerprints provide a solid theoretical framework for predicting cultivar-specific organoleptic profiles.

High concentrations of individual secoiridoids also strengthen the functional interest of the collection. Oleocanthal was originally identified through ibuprofen-like cyclooxygenase inhibition, and oleacein and oleocanthal have subsequently been associated with antioxidant activity and oil stability [47,48,49]. The present results identify oils suitable for future nutritional, storage and sensory experiments, particularly important for ‘Managjel FV’, whose very high oleocanthal content coexisted with a relatively polyunsaturated fatty-acid composition, and for ‘U.H. Himare’, which combined abundant dialdehydic secoiridoids with high oil content.

Every cultivar mean exceeded 250 mg/kg for the direct sum of hydroxytyrosol, tyrosol, and the six quantified secoiridoid derivatives—the cut-off value proposed in the literature as a screening proxy for the EFSA health claim [15,50]. This is encouraging in relation to the authorized statement that olive oil polyphenols contribute to the protection of blood lipids from oxidative stress. The EFSA health claim legal condition is at least 5 mg of hydroxytyrosol and its derivatives per 20 g of oil, together with the specified consumer information [35].

As the most highlighted cultivars, ‘Managjel FV’, ‘U.H. Himare’ and ‘Ulliri i Kuq’ had EFSA-related phenolic sums above 600 mg/kg, whereas even ‘U.K. Berat’, the lowest cultivar, averaged 293.5 mg/kg. Retail surveys have found that only a minority of oils exceed the corresponding compositional benchmark, illustrating its potential value for product differentiation [50].

### 4.3. Fatty-Acid Composition and Its Potential Role in Oxidative Stability

Oleic acid predominated in all oils, but its 14.6 percentage-point range separated two clear fatty-acid chemotype cultivars. ‘Unafka’ combined 79.92% oleic acid with 4.88% linoleic acid and the highest C18:1/C18:2 ratio (16.39), whereas ‘Kotruvsi’ and ‘Managjel FV’ had approximately 65% oleic acid and much higher linoleic acid, producing ratios of 3.89 and 4.89. The very large cultivar effect sizes are consistent with multi-environment evidence showing that genotype is usually the main source of fatty-acid variation, although season, temperature and genotype-by-environment interaction remain significant [10,11,22]. Similar work has discriminated olive cultivars primarily through oleic, linoleic and palmitic acids, confirming that these major fatty acids carry substantial cultivar (genetic) information [37].

This fatty-acid divergence mirrors the broad variability observed across Mediterranean germplasms [10,11]. The oleic acid content of ‘Unafka’ (79.92%) places it alongside major high-oleic international benchmarks such as ‘Picual’ (75–82%) and ‘Cornicabra’, whereas the lower oleic and higher linoleic proportions of ‘Kotruvsi’ (65.36%) and ‘Managjel FV’ (65.65%) resemble cultivars with moderate monounsaturated profiles, such as ‘Arbequina’ (60–68%) or ‘Empeltre’.

The fatty-acid correlation result in this study situates the expected oleic–linoleic negative correlation within a broader metabolic context that, although exploratory, aligns well with known biosynthetic pathways. Underlying the strongly negative oleic–linoleic correlation is microsomal oleate desaturation: oleic acid (C18:1) is converted to linoleic acid (C18:2) by endoplasmic-reticulum Δ12-desaturases. In olive, the genes OeFAD2-2 and OeFAD2-5, together with the substrate preference of extraplastidial acyltransferases, have been identified as the main determinants of the mesocarp C18:1/C18:2 ratio [51]. Upstream of this step, stearoyl-ACP desaturase (SAD) enzymes catalyse the first desaturation—stearic acid (C18:0) to oleic acid—and their transcript levels vary with cultivar [52], as well as with abiotic factors such as temperature and darkness [53]. Moving toward longer saturated chains, the positive correlations linking stearic (C18:0), arachidic (C20:0), behenic (C22:0) and lignoceric (C24:0) acids are consistent with the stepwise action of fatty acid elongase (FAE) complexes, which reside in the endoplasmic reticulum and add successive two-carbon units to long-chain acyl-CoAs [54]. Finally, the correlation between margaric (C17:0) and margaroleic (C17:1) acids suggests that a fraction of the odd-chain C17 fatty-acid pool undergoes desaturation.

These mechanisms offer plausible explanations for the ‘Unafka’ and ‘Kotruvsi’ profiles. However, in this study no enzyme expression, acyl flux or isotope tracing was measured; therefore, the observed correlation should be read as a source of pathway hypotheses rather than evidence that a particular enzyme, allele or reaction caused each cultivar position.

The C18:1/C18:2 ratio provides a useful indication of lipid susceptibility to oxidation, not a measured shelf-life value. Rancimat studies identify linoleic acid as a dominant negative determinant of oxidative stability and show that phenols are critical co-predictors, especially in oils with intermediate-to-high polyunsaturation [16,55,56]. Multi-genotype trials demonstrate that fatty-acid composition and phenolic antioxidants jointly determine oxidative and sensory performance, and that location modulates their expression [17]. A recent study of 80 Greek olive cultivars revealed a clear association between MUFA abundance and the capacity of olive oil to preserve phenolic integrity. Accelerated oxidation experiments showed that oils with higher MUFA content exhibited slower degradation of oleocanthal and oleacein, supporting a protective effect of a MUFA-rich, oleic acid-dominated matrix on phenolic stability [57]. These findings support ‘Unafka’, ‘U.K. Berat’ and ‘Kalinjot’ as candidates for high intrinsic oxidative resistance, but confirmation requires direct induction-time and storage measurements.

Conversely, the phenol-rich but lower-oleic oils illustrate why a single quality marker is insufficient. ‘Managjel FV’ had the highest total phenolics and oleocanthal but also the highest palmitic acid and one of the lowest oleic/linoleic ratios. ‘Kotruvsi’ similarly combined high phenolics and luteolin with the most linoleic-rich profile. In monovarietal comparisons, high phenolic content does not always offset a linoleic-rich fatty-acid matrix, and the balance between the two is both cultivar- and processing-dependent [49,56]. These cultivars should therefore be described as chemically distinctive rather than globally superior or inferior until oxidative stability, volatile evolution and sensory quality are measured together.

### 4.4. Cultivar Oil Content and Multi-Trait Quality Value

The corrected fresh-matter oil content ranged from 22.2% in ‘U.K. Berat’ to 29.9% in ‘U.H. Himare’, while dry-matter oil content ranged from 47.5% to 55.0%. These values refer to fruit oil content, not industrial extraction efficiency; actual mill recovery also depends on paste rheology, moisture, malaxation and equipment. Cultivar, ripening and harvest timing can modify oil accumulation and the balance between quantity and compositional quality [58,59]. The common maturity and processing conditions used here make the ranking useful for this harvest, but do not establish a permanent production hierarchy.

Several multi-trait combinations deserve follow-up. ‘U.H. Himare’ and ‘Ulliri i Kuq’ combined high fresh- and dry-matter oil contents with high phenolic sums, although their oleic/linoleic ratios were moderate. ‘Karen’ offered the most balanced profile relative to the collection medians, with high phenolics, a comparable favourable oleic/linoleic ratio and high fresh-matter oil content. ‘Unafka’ maximized the oleic/linoleic ratio but had intermediate phenolics and oil content, while ‘U.K. Berat’ combined high oleic acid with the lowest phenolic and oil contents. These contrasts show that selection for Albanian monovarietal oil production should use an explicit breeding or market objective rather than a universal ranking.

### 4.5. Global Quality Evaluation and Albanian Monovarietal Oils Valorisation

The multivariate analyses confirmed that phenolics, fatty acids and technological traits provide complementary information. Phenolic HCA retained four groups, fatty-acid HCA retained two groups along the oleic-linoleic gradient, and the joint matrix (phenols, fatty acids, and technological parameters) retained three groups. Comparable studies have differentiated cultivars, harvest periods and protected-origin oils using phenolic fingerprints and multivariate models [19,60,61].

The joint solution provides a useful interpretation of multi-trait strategies. Cluster 1 represented the most oleic and least phenolic profile (75.45% oleic acid; 351.2 mg kg^−1^ total phenolics; 349.4 mg kg^−1^ health-claim-related sum) and the lowest dry-matter oil content (49.80%), although ‘Pulazeqin’ was an aglycone-rich exception. Cluster 2 paired ‘Karen’ and ‘U.H. Himare’ and combined high phenolics (622.9 mg kg^−1^), high oleocanthal and oleacein, intermediate oleic acid (70.96%) and the highest dry-matter oil content (53.97%). It can therefore be described as EDA-rich across both the ligstroside-derived and oleuropein-derived families. Cluster 3 grouped ‘Kotruvsi’, ‘Managjel FV’ and ‘Ulliri i Kuq’ and combined the highest total and health-claim-related phenolics (693.7 and 646.6 mg kg^−1^), high oleuropein and ligstroside aglycones, the lowest oleic acid (66.19%) and intermediate-high dry-matter oil content (51.70%), giving a relatively aglycone-rich profile across both families. PCA therefore distinguished complementary profiles, not globally superior classes: PC1 opposed oleic-rich to phenol- and oil-associated traits, whereas PC2 separated aglycone/hydroxytyrosol/luteolin expression from oleacein/oleocanthal and dry-matter oil content. These family and molecular-form descriptors summarize cultivar profiles; they do not establish pathway flux or causal metabolic trade-offs.

The variability of phenolic compounds observed in this study is consistent with previous reports showing substantial cultivar-dependent chemical diversity in Albanian olive oils. Comparative phenolic profiling studies demonstrated marked differences among Albanian monovarietal oils and showed that both cultivar identity and geographical origin contribute to sample differentiation [29], while broader reviews have documented significant variation in fatty-acid composition, phenolic composition and volatile profiles across Albanian cultivars and production regions [30].

The chemical diversity is consistent with, although independent from, the documented genetic richness of Albanian olives. A nationwide SSR survey identified 183 unique genotypes among 194 accessions and described Albanian native germplasm as a distinctive genetic resource represented by a highly informative core collection [24]. Mediterranean-wide analyses nevertheless indicate that olive diversity is structured more as a continuum than as a set of genetically isolated regional lineages. Using 25 SSR loci, Díez et al. (2015) identified three broadly western, central and eastern Mediterranean ancestry groups together with numerous admixed cultivars, concluding that admixture was a major driver of olive diversification [27]. A more recent parentage atlas based on 96 EST-SNPs recovered a similar three-group framework but classified 58% of 853 accessions as admixed, further highlighting the importance of historical gene flow across the Mediterranean Basin [28].

The singular position of ‘Kotruvsi’ is particularly noteworthy in this context. Gómez-Gálvez et al. (2026) [28] inferred ‘Toffehi Tataouine’ and ‘Safrawi’ as the most likely parental pair of this Albanian cultivar, placing it within a pedigree network that spans multiple Mediterranean regions. Thus, while ‘Kotruvsi’ belongs to a germplasm pool regarded as genetically distinctive within Albania, its inferred ancestry also illustrates the broader historical connectivity linking eastern, central and western Mediterranean olive resources.

From an applied perspective, these cultivar-specific fingerprints can support authenticity, Digital Product Passport (DPP), cultivar selection and differentiated Albanian products. Phenolic profiles have successfully discriminated Spanish Protected Designation of Origin (PDO) oils, demonstrating their potential as part of an origin-control system when supported by validated sampling and classification models [60]. Recent characterisation of Kalinjot oils likewise identified a basis for geographical valorisation, but also showed lot-to-lot variation in phenols, volatiles and sensory attributes [31]. Consumer research confirms that origin, sensory properties and credence attributes such as geographical indications and sustainability claims influence olive oil preferences and can control premium prices, although their effectiveness depends on clear communication and the coexistence of verified intrinsic quality [62]. For Albania, the present profiles are a strong discovery dataset; a PDO/PGI specification would additionally require multi-year reference ranges, traceability, standardised processing, sensory description and independent validation.

This study provides an important overview within main cultivar variation, but all observations derive from one site, one maturity index and one crop season. Consequently, the single crop-year evaluation constitutes an inherent limitation of this baseline study, as interannual climatic variations (e.g., temperature fluctuations and precipitation patterns during fruit development) are well known to modulate minor compound accumulation and fatty-acid desaturation. Multi-year and multi-environment trials across contrasting Albanian growing regions will therefore be essential to delineate stable genotypic traits from plastic environmental responses. Future research will build upon this initial baseline by integrating multi-year sampling with broader metabolomic profiling, encompassing volatile organic compounds, tocopherols, and triterpenic acids, alongside official organoleptic panel tests [11,22,63]. Also, future work on Albanian olive cultivars should combine Rancimat or storage trials with volatile analysis and a sensory evaluation panel test. Sampling the same authenticated trees across maturity stages, years and contrasting Albanian environments would separate stable cultivar effects from plastic responses. Integrating those phenotypes with dense SNP data could then test whether the distinctive profiles of ‘Kotruvsi’, ‘Managjel FV’, ‘U.H. Himare’, ‘Ulliri i Kuq’ and ‘Unafka’ are associated with ancestry or candidate metabolic loci. This staged validation is essential before recommending fixed cultivar rankings, shelf-life claims or protected-origin specifications.

Overall, our study demonstrates that Albanian olive germplasm contains substantial and practically relevant differentiation in oil phenolics, fatty-acid composition and technological traits. ‘Managjel FV’ combined the highest total phenolics with oleocanthal dominance; ‘U.H. Himare’ combined high EDA derivatives from both the oleuropein- and ligstroside-related families (oleacein and oleocanthal) with high dry-matter oil content; ‘Pulazeqin’ represented an oleuropein aglycone/oleomissional-rich profile; ‘Ulliri i Kuq’ was distinguished by ligstroside aglycone and tyrosol; ‘Kotruvsi’ by luteolin and hydroxytyrosol; and ‘Unafka’ by the most favourable oleic/linoleic balance. The chemometric groups capture complementary compositional strategies rather than a single hierarchy of quality. Together with verified cultivar identity and multi-environment validation, these profiles can support conservation, breeding and differentiated valorisation of Albanian olive genetic resources.

## 5. Conclusions

This study provides an integrated characterisation of the phenolic, fatty-acid and technological diversity of 11 authenticated Albanian olive cultivars evaluated under common field, maturity and extraction conditions. The significant cultivar effects and the broad compositional ranges demonstrate that Albania’s still incompletely characterised olive genetic resources contain substantial quality variation of direct value for conservation and use. Total quantified phenolics varied 2.7-fold, from 296.5 mg kg^−1^ in ‘U.K. Berat’ to 791.0 mg kg^−1^ in ‘Managjel FV’. Importantly, high totals represented different fingerprints: ‘Managjel FV’ was dominated by oleocanthal; ‘U.H. Himare’ combined oleocanthal and oleacein with the highest dry-matter oil content; ‘Pulazeqin’ was rich in oleuropein aglycone and oleomissional; ‘Ulliri i Kuq’ in ligstroside aglycone and tyrosol; and ‘Kotruvsi’ in luteolin and hydroxytyrosol. Fatty-acid composition was equally discriminating. Oleic acid ranged from 65.36% in ‘Kotruvsi’ to 79.92% in ‘Unafka’, while the oleic/linoleic ratio ranged from 3.89 to 16.39, identifying ‘Unafka’, ‘U.K. Berat’ and ‘Kalinjot’ as candidates for favourable intrinsic oxidative resistance that still requires direct stability testing.

Every cultivar mean exceeded the 250 mg kg^−1^ descriptive screening reference for the direct EFSA-related phenols, and ‘Managjel FV’, ‘U.H. Himare’ and ‘Ulliri i Kuq’ exceeded 600 mg kg^−1^. This result supports further health-oriented evaluation. The detailed chemical characterisation likewise identified complementary strategies for cultivar characterisation and classification, such as oleic-rich, EDA-rich and aglycone-rich monovarietal oil profiles, rather than a universal ranking, emphasizing that cultivar selection should match breeding, sensory, stability, yield or market objectives.

These compositional fingerprints can underpin authenticity tools, Digital Product Passport (DPP), targeted breeding and differentiated Albanian monovarietal oils, including the future development of PDO/PGI specifications. However, protected-origin ranges and commercial or health-related claims require traceability, standardised processing, sensory assessment and independent validation. This preliminary one-year characterisation study paves the way for a wider characterisation of these cultivars under multi-year and multi-environment trials across Albanian growing regions.

## Figures and Tables

**Table 1:** Absolute phenolic content of analysed cultivars and derived sums (mg kg^−1^ oil).

Cultivar	OLC	OLA	OLEA	OLM	LIGA	OLK	HTyr	LUT	API	Tyr	Total	EFSA Sum
Kalinjot	41.6 ± 1.1 f	72.6 ± 5.6 b	95.7 ± 4.2 d	19.9 ± 2.2 cde	47.0 ± 0.6 d	45.9 ± 0.7 bc	0.0 ± 0.0 d	1.0 ± 0.2 fg	0.0 ± 0.0 b	7.0 ± 0.4 g	330.7 ± 7.9 g	329.6 ± 7.9 g
Karen	302.3 ± 5.4 b	57.2 ± 8.9 c	113.2 ± 3.0 c	14.6 ± 1.9 ef	40.6 ± 0.9 f	22.7 ± 0.2 e	0.0 ± 0.0 d	33.4 ± 3.4 b	0.6 ± 0.8 a	19.7 ± 1.6 ef	604.3 ± 9.8 c	570.3 ± 9.8 c
Koran	36.8 ± 1.0 g	72.6 ± 11.2 b	134.4 ± 3.7 b	23.0 ± 4.0 c	40.0 ± 0.9 f	25.6 ± 0.3 e	0.0 ± 0.0 d	0.3 ± 0.1 h	0.0 ± 0.0 b	5.7 ± 0.2 g	338.5 ± 11.4 fg	338.2 ± 11.5 fg
Kotruvsi	189.1 ± 6.2 d	63.7 ± 6.9 bc	132.9 ± 7.4 b	16.2 ± 3.0 def	43.5 ± 0.9 e	31.3 ± 0.4 d	13.0 ± 1.0 a	79.6 ± 9.6 a	0.0 ± 0.0 b	79.9 ± 9.2 c	649.3 ± 23.7 b	569.6 ± 21.6 c
Managjel FV	411.7 ± 12.8 a	56.7 ± 5.8 c	163.5 ± 4.2 a	7.7 ± 1.3 g	47.5 ± 0.8 d	15.5 ± 0.1 f	2.2 ± 0.4 b	36.8 ± 3.6 b	0.0 ± 0.0 b	49.3 ± 4.9 d	791.0 ± 17.9 a	754.1 ± 18.6 a
Mixani	35.6 ± 4.9 g	45.3 ± 7.4 d	131.7 ± 13.2 b	34.6 ± 13.0 b	40.3 ± 1.5 f	51.4 ± 15.6 b	0.4 ± 0.6 d	0.9 ± 0.6 g	0.0 ± 0.0 b	10.7 ± 8.6 g	350.9 ± 15.6 ef	350.0 ± 15.2 ef
Pulazeqin	21.3 ± 0.6 h	30.6 ± 4.3 e	172.3 ± 5.1 a	46.0 ± 4.5 a	39.3 ± 0.8 f	42.6 ± 0.3 c	0.0 ± 0.0 d	1.7 ± 0.3 e	0.0 ± 0.0 b	8.8 ± 0.4 g	362.5 ± 8.1 e	360.9 ± 8.2 e
U.H. Himare	280.3 ± 9.3 c	183.0 ± 19.4 a	70.3 ± 2.7 f	12.4 ± 2.5 f	51.0 ± 0.9 c	31.9 ± 0.4 d	0.0 ± 0.0 d	1.5 ± 0.3 ef	0.0 ± 0.0 b	11.1 ± 0.7 fg	641.4 ± 19.4 b	640.0 ± 19.4 b
U.K. Berat	22.3 ± 0.5 h	33.1 ± 6.0 e	105.6 ± 3.6 c	20.3 ± 2.3 cd	42.5 ± 0.8 e	47.0 ± 0.5 bc	1.5 ± 0.2 c	3.1 ± 0.2 d	0.0 ± 0.0 b	21.2 ± 1.0 e	296.5 ± 5.0 h	293.5 ± 5.1 h
Ulliri i Kuq	139.5 ± 3.6 e	10.0 ± 0.5 f	88.1 ± 4.6 e	0.0 ± 0.0 i	205.8 ± 2.7 a	46.1 ± 0.6 bc	0.0 ± 0.0 d	24.9 ± 3.0 c	0.0 ± 0.0 b	126.3 ± 11.8 a	640.9 ± 11.0 b	616.0 ± 10.5 b
Unafka	137.2 ± 3.3 e	10.0 ± 0.3 f	55.3 ± 2.0 g	0.9 ± 0.7 h	67.6 ± 1.4 b	61.0 ± 0.4 a	0.0 ± 0.0 d	3.8 ± 0.5 d	0.0 ± 0.0 b	92.6 ± 7.0 b	428.2 ± 9.0 d	424.4 ± 9.0 d

Note: Values are means ± standard deviations (n = 10 independent samples) followed by Tukey compact-letter groupings. OLC, oleocanthal; OLA, oleacein; OLEA, oleuropein aglycone; OLM, oleomissional; LIGA, ligstroside aglycone; OLK, oleokoronal; HTyr, hydroxytyrosol; LUT, luteolin; API, apigenin; Tyr, tyrosol; EFSA, European Food Safety Authority.

**Table 2:** Key phenolic, fatty-acid and technological traits of the 11 olive cultivars.

Cultivar	Total Phenolics (mg kg^−1^)	EFSA-Related Sum (mg kg^−1^)	Oleic Acid (%)	Linoleic Acid (%)	Oleic/Linoleic ratio	Moisture (%)	Oil, FW (%)	Oil, DM (%)
Kalinjot	330.7 ± 7.9 g	329.6 ± 7.9 g	76.28 ± 0.39 b	9.65 ± 0.25 e	7.91 ± 0.23 cd	47.5 ± 2.3 cd	28.1 ± 1.5 ab	53.5 ± 2.4 abc
Karen	604.3 ± 9.8 c	570.3 ± 9.8 c	73.91 ± 0.37 d	8.94 ± 0.25 f	8.28 ± 0.26 c	47.2 ± 2.2 d	28.0 ± 2.0 ab	53.0 ± 2.6 abcd
Koran	338.5 ± 11.4 fg	338.2 ± 11.5 fg	75.04 ± 0.27 c	9.25 ± 0.19 ef	8.12 ± 0.20 cd	50.9 ± 2.3 abc	24.3 ± 1.4 cde	49.5 ± 1.8 cde
Kotruvsi	649.3 ± 23.7 b	569.6 ± 21.6 c	65.36 ± 0.39 h	16.80 ± 0.28 a	3.89 ± 0.08 i	47.3 ± 5.4 cd	27.3 ± 3.9 abc	51.7 ± 5.0 abcde
Managjel FV	791.0 ± 17.9 a	754.1 ± 18.6 a	65.65 ± 0.42 h	13.42 ± 0.30 b	4.89 ± 0.14 h	52.1 ± 2.4 ab	23.3 ± 2.1 de	48.7 ± 2.6 de
Mixani	350.9 ± 15.6 ef	350.0 ± 15.2 ef	73.17 ± 0.77 e	9.42 ± 0.59 e	7.80 ± 0.55 d	52.4 ± 1.0 ab	23.2 ± 1.1 de	48.7 ± 1.7 de
Pulazeqin	362.5 ± 8.1 e	360.9 ± 8.2 e	71.84 ± 0.23 f	10.26 ± 0.19 d	7.00 ± 0.15 e	48.8 ± 2.2 bcd	25.4 ± 2.0 bcd	49.5 ± 3.0 cde
U.H. Himare	641.4 ± 19.4 b	640.0 ± 19.4 b	68.01 ± 0.37 g	11.24 ± 0.18 c	6.05 ± 0.12 f	45.6 ± 1.9 d	29.9 ± 2.0 a	55.0 ± 2.5 a
U.K. Berat	296.5 ± 5.0 h	293.5 ± 5.1 h	76.45 ± 0.28 b	8.48 ± 0.12 g	9.02 ± 0.15 b	53.3 ± 2.3 a	22.2 ± 2.2 e	47.5 ± 4.3 e
Ulliri i Kuq	640.9 ± 11.0 b	616.0 ± 10.5 b	67.57 ± 0.33 g	13.14 ± 0.23 b	5.14 ± 0.11 g	46.7 ± 1.8 d	29.2 ± 2.6 a	54.7 ± 4.1 ab
Unafka	428.2 ± 9.0 d	424.4 ± 9.0 d	79.92 ± 0.33 a	4.88 ± 0.16 h	16.39 ± 0.59 a	51.8 ± 1.8 ab	24.1 ± 0.8 cde	50.0 ± 1.5 bcde

Note: Values are means ± standard deviations (n = 10 independent samples). Within each column, means sharing at least one lowercase letter were not significantly different according to Tukey’s honestly significant difference test on the selected transformed scale (*p* < 0.05). European Food Safety Authority (EFSA)-related sum denotes the direct mass sum used as a screening index. FW, fresh-matter basis; DM, dry-matter basis.

**Table 3:** Fatty-acid composition of analysed cultivars (% of total identified fatty acids) and oleic/linoleic acid ratio.

Cultivar	C16:0	C16:1	C17:0	C17:1	C18:0	C18:1	C18:2	C18:3	C20:0	C20:1	C22:0	C23:0	C24:0	C18:1/C18:2
Kalinjot	9.75 ± 0.22 i	0.17 ± 0.00 i	0.04 ± 0.00 f	0.07 ± 0.00 e	2.90 ± 0.06 d	76.28 ± 0.39 b	9.65 ± 0.25 e	0.43 ± 0.01 d	0.26 ± 0.01 c	0.25 ± 0.00 b	0.13 ± 0.00 f	0.01 ± 0.00 f	0.07 ± 0.00 c	7.91 ± 0.23 cd
Karen	11.99 ± 0.17 f	0.98 ± 0.02 b	0.01 ± 0.00 h	0.03 ± 0.00 f	3.02 ± 0.05 c	73.91 ± 0.37 d	8.94 ± 0.25 f	0.44 ± 0.01 cd	0.27 ± 0.01 c	0.19 ± 0.01 f	0.13 ± 0.00 e	0.01 ± 0.00 c	0.07 ± 0.00 c	8.28 ± 0.26 c
Koran	10.66 ± 0.16 gh	0.15 ± 0.00 i	0.11 ± 0.00 a	0.18 ± 0.01 a	3.24 ± 0.06 b	75.04 ± 0.27 c	9.25 ± 0.19 ef	0.53 ± 0.01 a	0.33 ± 0.01 b	0.26 ± 0.00 a	0.16 ± 0.00 c	0.01 ± 0.00 c	0.08 ± 0.00 b	8.12 ± 0.20 cd
Kotruvsi	13.99 ± 0.26 d	0.40 ± 0.01 f	0.06 ± 0.00 d	0.17 ± 0.00 b	2.12 ± 0.04 g	65.36 ± 0.39 h	16.80 ± 0.28 a	0.54 ± 0.01 a	0.17 ± 0.00 f	0.21 ± 0.00 e	0.12 ± 0.00 h	0.01 ± 0.00 d	0.07 ± 0.00 d	3.89 ± 0.08 i
Managjel FV	16.25 ± 0.22 a	0.67 ± 0.02 d	0.05 ± 0.00 e	0.16 ± 0.00 c	2.71 ± 0.03 e	65.65 ± 0.42 h	13.42 ± 0.30 b	0.45 ± 0.01 c	0.22 ± 0.01 e	0.20 ± 0.00 f	0.14 ± 0.00 d	0.01 ± 0.00 g	0.08 ± 0.00 b	4.89 ± 0.14 h
Mixani	12.05 ± 0.41 f	0.25 ± 0.05 h	0.08 ± 0.02 b	0.13 ± 0.01 d	3.62 ± 0.16 a	73.17 ± 0.77 e	9.42 ± 0.59 e	0.35 ± 0.01 f	0.38 ± 0.02 a	0.23 ± 0.01 c	0.20 ± 0.01 a	0.01 ± 0.00 c	0.10 ± 0.00 a	7.80 ± 0.55 d
Pulazeqin	12.80 ± 0.15 e	0.91 ± 0.03 c	0.05 ± 0.00 de	0.17 ± 0.00 b	3.01 ± 0.06 c	71.84 ± 0.23 f	10.26 ± 0.19 d	0.37 ± 0.01 e	0.25 ± 0.01 d	0.15 ± 0.00 h	0.12 ± 0.00 h	0.01 ± 0.00 e	0.05 ± 0.00 f	7.00 ± 0.15 e
U.H. Himare	15.81 ± 0.25 b	1.28 ± 0.02 a	0.01 ± 0.00 h	0.03 ± 0.00 f	2.50 ± 0.06 f	68.01 ± 0.37 g	11.24 ± 0.18 c	0.47 ± 0.01 b	0.25 ± 0.00 d	0.15 ± 0.00 h	0.16 ± 0.00 c	0.01 ± 0.00 a	0.07 ± 0.00 c	6.05 ± 0.12 f
U.K. Berat	10.52 ± 0.19 h	0.15 ± 0.00 i	0.08 ± 0.00 bc	0.13 ± 0.00 d	3.00 ± 0.04 c	76.45 ± 0.28 b	8.48 ± 0.12 g	0.47 ± 0.01 b	0.28 ± 0.00 c	0.24 ± 0.01 b	0.12 ± 0.00 g	0.00 ± 0.00 h	0.08 ± 0.00 b	9.02 ± 0.15 b
Ulliri i Kuq	14.56 ± 0.15 c	0.46 ± 0.01 e	0.07 ± 0.00 c	0.17 ± 0.00 b	2.92 ± 0.03 cd	67.57 ± 0.33 g	13.14 ± 0.23 b	0.48 ± 0.01 b	0.24 ± 0.01 d	0.18 ± 0.00 g	0.13 ± 0.00 ef	0.01 ± 0.00 b	0.06 ± 0.00 e	5.14 ± 0.11 g
Unafka	10.96 ± 0.19 g	0.33 ± 0.01 g	0.01 ± 0.00 g	0.03 ± 0.00 f	2.70 ± 0.05 e	79.92 ± 0.33 a	4.88 ± 0.16 h	0.37 ± 0.01 e	0.33 ± 0.01 b	0.22 ± 0.01 d	0.17 ± 0.00 b	0.01 ± 0.00 b	0.07 ± 0.00 c	16.39 ± 0.59 a

Note: Values are means ± standard deviations (n = 10 independent samples) followed by Tukey compact-letter groupings. Fatty acids are abbreviated by carbon number and degree of unsaturation. The C18:1/C18:2 column is a dimensionless derived ratio. Abbreviations: C16:0, palmitic acid; C16:1, palmitoleic acid; C17:0, margaric acid; C17:1, margaroleic acid; C18:0, stearic acid; C18:1, oleic acid; C18:2, linoleic acid; C18:3, linolenic acid; C20:0, arachidic acid; C20:1, gadoleic acid; C22:0, behenic acid; C23:0, tricosanoic acid; C24:0, lignoceric acid; C18:1/C18:2, oleic/linoleic acid ratio.

## Data Availability

The original contributions presented in this study are included in the article/Supplementary Materials. Further inquiries can be directed to the corresponding author.

## Supplementary Materials

The following supporting information can be downloaded at https://www.mdpi.com/article/10.3390/foods15203726/s1: Figure S1. Relative distribution of the 10 quantified phenolic compounds within each cultivar. Stacked bars show compound percentages of total quantified phenolics; Figure S2. Exploratory Pearson correlation matrix among the 10 individual phenolics, calculated across the 110 sample-level oils. Circle colour indicates the sign, circle area scales with the absolute Pearson correlation coefficient (*r*). Only associations that remained significant after Benjamini–Hochberg false-discovery-rate correction are displayed: * q < 0.05, ** q < 0.01 and *** q < 0.001; blank off-diagonal cells were not significant (q ≥ 0.05); Figure S3. Relative fatty-acid composition of the 11 monovarietal oils. Stacked bars show the mean percentage of each of the 13 identified fatty acids (n = 10 independent oil samples per cultivar); Figure S4. Bivariate screening of the EFSA-related phenolic compounds against the oleic/linoleic acid ratio. Points show cultivar means, dashed reference lines show the collection medians and define descriptive quadrants only; they are not regulatory or quality-classification thresholds; Figure S5. Exploratory Pearson correlation matrix between the 10 individual phenolics, total quantified phenolics and 13 fatty-acid percentages. Circle colour indicates the sign and circle area scales with the absolute Pearson correlation coefficient (r). Only associations that remained significant after Benjamini–Hochberg false-discovery-rate correction are displayed: * q < 0.05, ** q < 0.01 and *** q < 0.001; blank cells were not significant (q ≥ 0.05); Figure S6. Fruit technological traits by cultivar: (a) moisture, (b) total solvent-extractable oil content on a fresh-matter basis and (c) total solvent-extractable oil content on a dry-matter basis. Bars show means and error bars show standard deviations (n = 10 independent samples). Within each panel, means sharing at least 1 lowercase letter were not significantly (p < 0.05); Figure S7. Joint chemical-technological chemometric cultivar clustering. (a) Principal component analysis score-loading biplot of the 10 phenolics, 13 fatty acids and 3 technological variables. Points represent independent sample-level oils; point symbols identify cultivars and colours identify the 3 joint hierarchical cluster analysis groups. Large cultivar symbols mark cultivar centroids, stars mark cluster centroids and shaded regions are 95% covariance ellipses. All 26 loading vectors are displayed and coloured by block: phenolics (purple), fatty acids (orange) and technological traits (blue). (b) Ward-linkage dendrogram of z-standardised cultivar means; the red dashed line shows the cut-off defining 3 clusters. Branch colours match panel (a); Figure S8. Exploratory Pearson correlation matrix among the 13 fatty-acid percentages across 110 independent sample-level oils. Circle colour indicates the sign of Pearson’s correlation coefficient (*r*), circle size indicates the correlation scale in numerical level (*r*). Only associations that remained significant after Benjamini–Hochberg false-discovery-rate correction are displayed: * q < 0.05, ** q < 0.01 and *** q < 0.001; blank off-diagonal cells were not significant (q ≥ 0.05), and diagonal cells equal 1.00; Table S1: Simple sequence repeat-authenticated cultivar designations and names used in the manuscript; Table S2: Liquid chromatography–tandem mass spectrometry multiple-reaction-monitoring conditions used for phenolic determination; Table S3: Matrix-matched calibration models used for absolute phenolic quantification; Table S4. One-way ANOVA testing the effect of cultivar on each analytical response; Table S5. Cultivar membership and bootstrap support of the exploratory chemometric clusters; Table S6. Silhouette comparison and bootstrap stability of the exploratory hierarchical cluster analysis solutions; Table S7. PCA loadings and variable contributions to the 2 displayed principal components for (A) phenolics, (B) fatty acids and (C) the joint chemical-technological matrix.

## Abbreviations

The following abbreviations are used in this manuscript: AADF, Albanian-American Development Foundation; ANOVA, analysis of variance; DM, dry matter; EFSA, European Food Safety Authority; EVOO, extra virgin olive oil; FAME, fatty-acid methyl ester; FW, fresh matter; GC-FID, gas chromatography with flame-ionisation detection; HCA, hierarchical cluster analysis; HSD, honestly significant difference; IOC, International Olive Council; LC-MS/MS, liquid chromatography–tandem mass spectrometry; MRM, multiple-reaction monitoring; MUFA, monounsaturated fatty acid; PCA, principal component analysis; PC1, first principal component; PC2, second principal component; B, mean within-cluster bootstrap co-membership; PDO, Protected Designation of Origin; PGI, Protected Geographical Indication; QTTB, Agricultural Technology Transfer Centre (ATTC); READ, Research Expertise from the Academic Diaspora; SNP, single-nucleotide polymorphism; SSR, simple sequence repeat; UCO, Universidad de Córdoba; VOO, virgin olive oil.
