# Supplementary material for: Cultivar-Specific Phenolic and Fatty-Acid Composition of Albanian Monovarietal Olive Oils
Source: Foods. 2026 Oct 19;15(20):3726. doi: 10.3390/foods15203726 (PMC13704448; doi:10.3390/foods15203726)
Supplement: Supplementary file 1 [file foods-15-03726-s001.zip › Supplementary_Figures_09_09_2026.pdf]

Article

Cultivar-specific phenolic and fatty-acid composition of Albanian monovarietal olive oils

Fatjon Hoxha[1†], Mivan Peçi[2†], Concepción Muñoz Díez[3], Feliciano Priego Capote[4], Adhurim Lazaj[5], Anila Kopali[1], Elisa Peçi[6], Onejda Kyçyk[7] and Hristofor Miho[3*]

Supplementary Figures

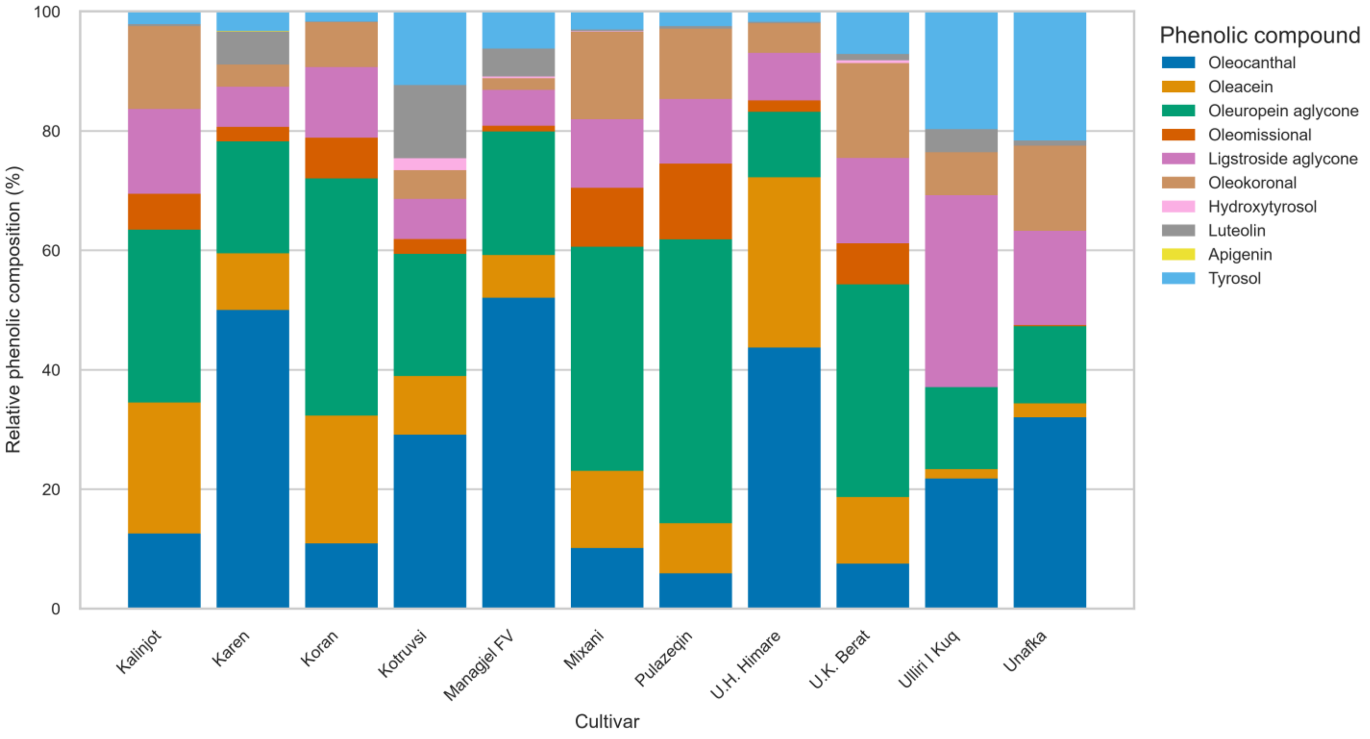

Figure S1. Relative distribution of the 10 quantified phenolic compounds within each cultivar. Stacked bars show compound percentages of total quantified phenolics.

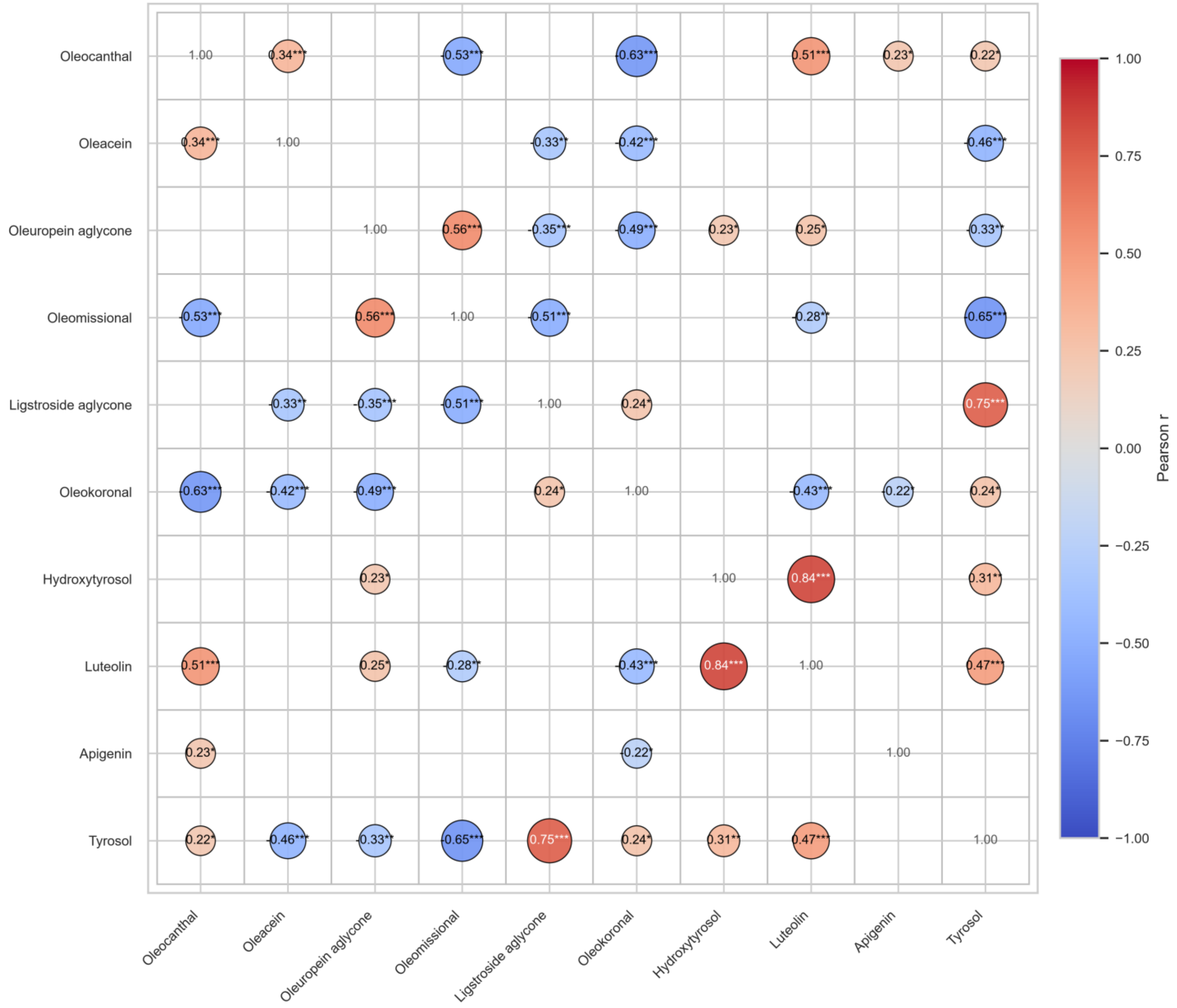

Figure S2. Exploratory Pearson correlation matrix among the 10 individual phenolics, calculated across the 110 sample-level oils. Circle colour indicates the sign, circle area scales with the absolute Pearson correlation coefficient (r). Only associations that remained significant after Benjamini–Hochberg false-discovery-rate correction are displayed: * $q < 0.05$, ** $q < 0.01$ and *** $q < 0.001$; blank off-diagonal cells were not significant ($q \geq 0.05$).

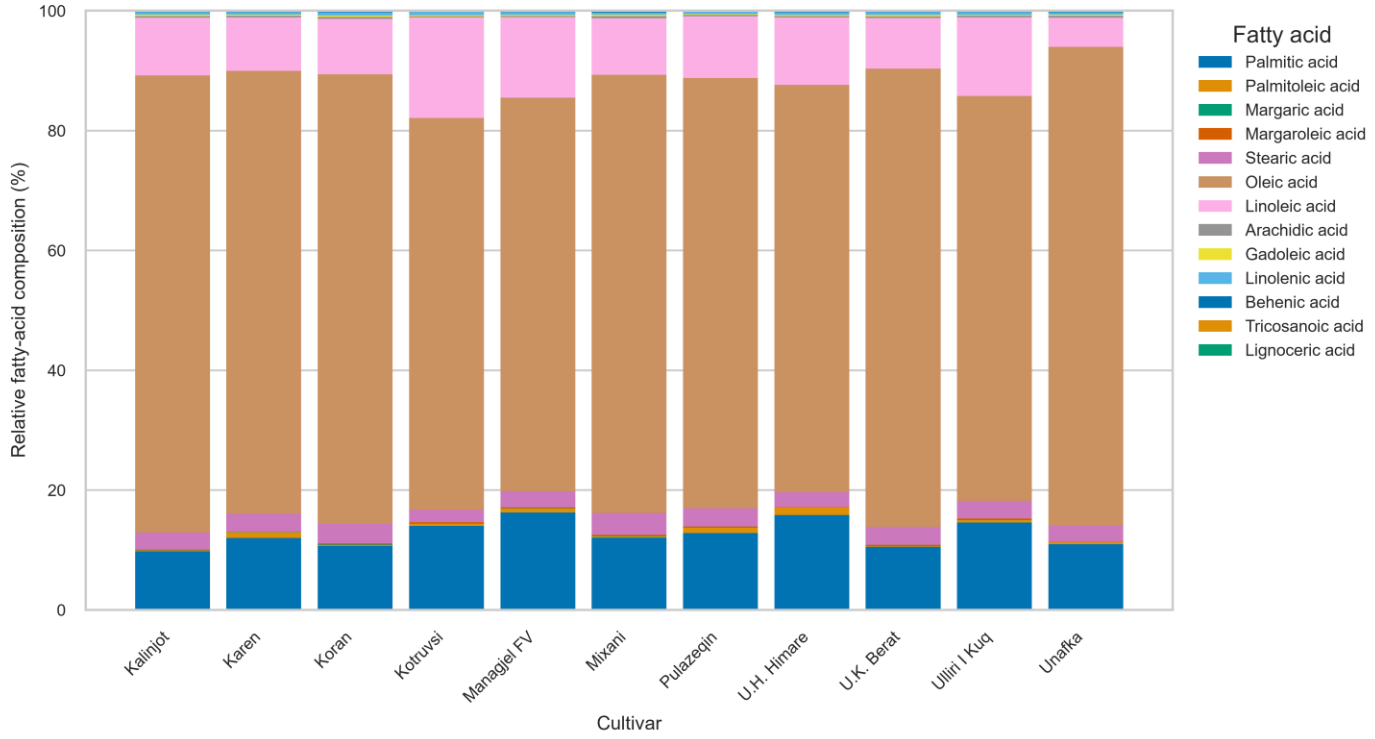

Figure S3. Relative fatty-acid composition of the 11 monovarietal oils. Stacked bars show the mean percentage of each of the 13 identified fatty acids (n = 10 independent oil samples per cultivar).

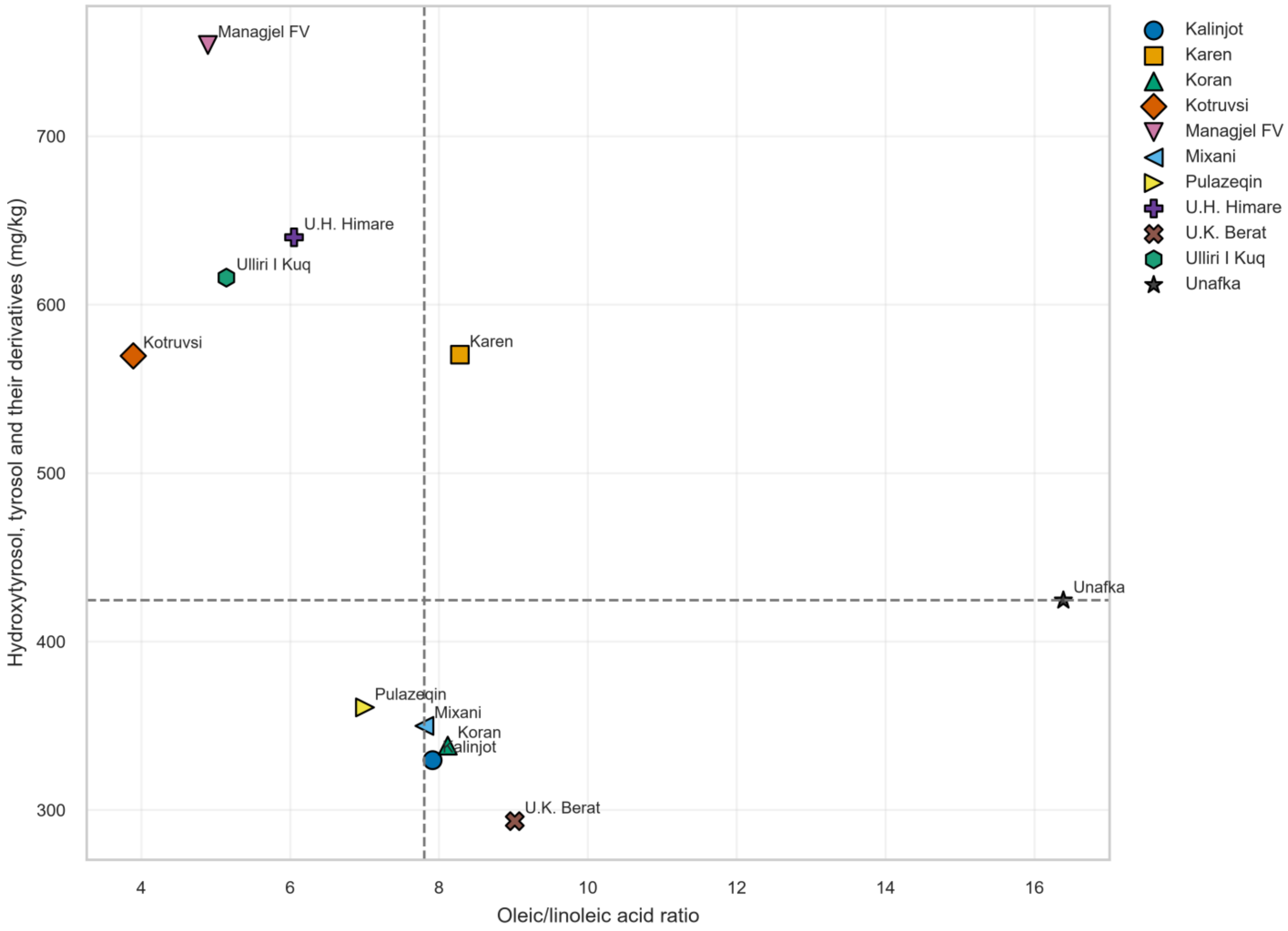

Figure S4. Bivariate screening of the EFSA-related phenolic compounds against the oleic/linoleic acid ratio. Points show cultivar means, dashed reference lines show the collection medians and define descriptive quadrants only; they are not regulatory or quality-classification thresholds.

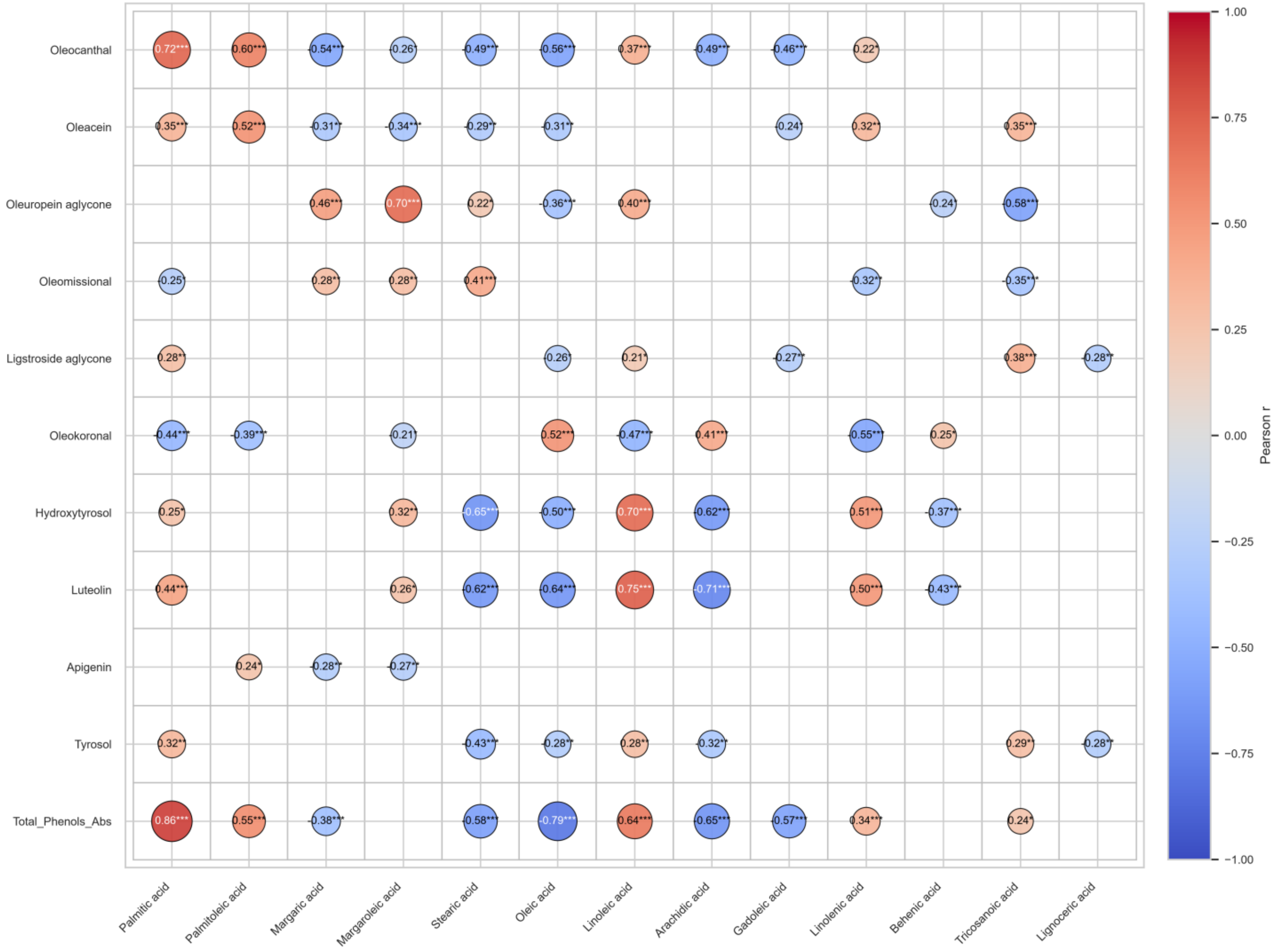

Figure S5. Exploratory Pearson correlation matrix between the 10 individual phenolics, total quantified phenolics and 13 fatty-acid percentages. Circle colour indicates the sign and circle area scales with the absolute Pearson correlation coefficient (*r*). Only associations that remained significant after Benjamini–Hochberg false-discovery-rate correction are displayed: * $q < 0.05$, ** $q < 0.01$ and *** $q < 0.001$; blank cells were not significant ($q \geq 0.05$). Abbreviations: FDR, false discovery rate; Total_Phenols_Abs, total quantified phenolics on the absolute concentration scale.

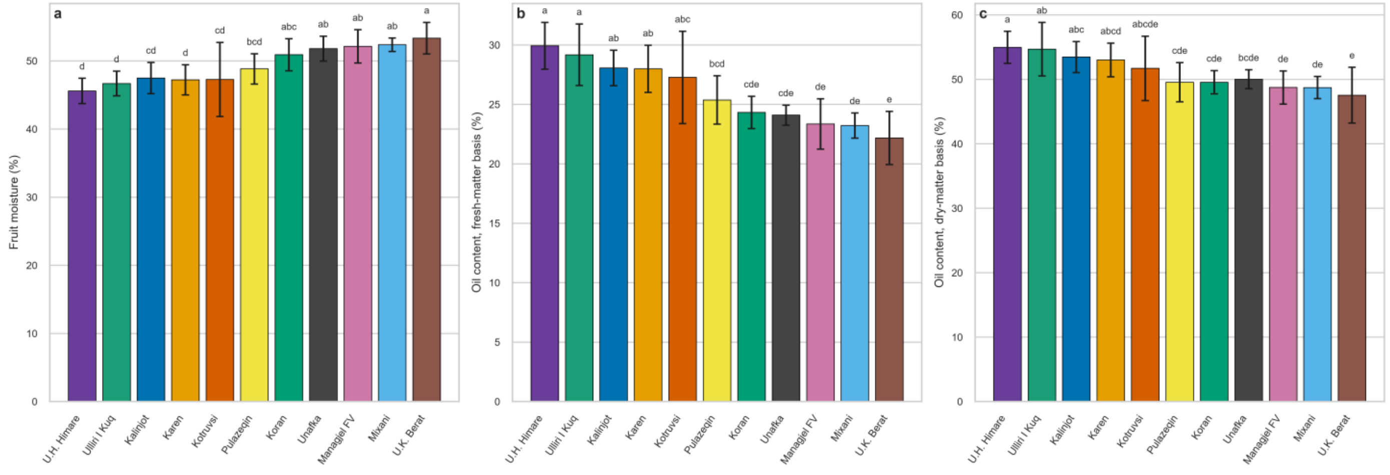

Figure S6. Fruit technological traits by cultivar: (a) moisture, (b) total solvent-extractable oil content on a fresh-matter basis and (c) total solvent-extractable oil content on a dry-matter basis. Bars show means and error bars show standard deviations (n = 10 independent samples). Within each panel, means sharing at least 1 lowercase letter were not significantly (p < 0.05). Abbreviations:

Oil_FW, oil content on a fresh-matter basis; Oil_DM, oil content on a dry-matter basis; FW, fresh-matter basis; DM, dry-matter basis.

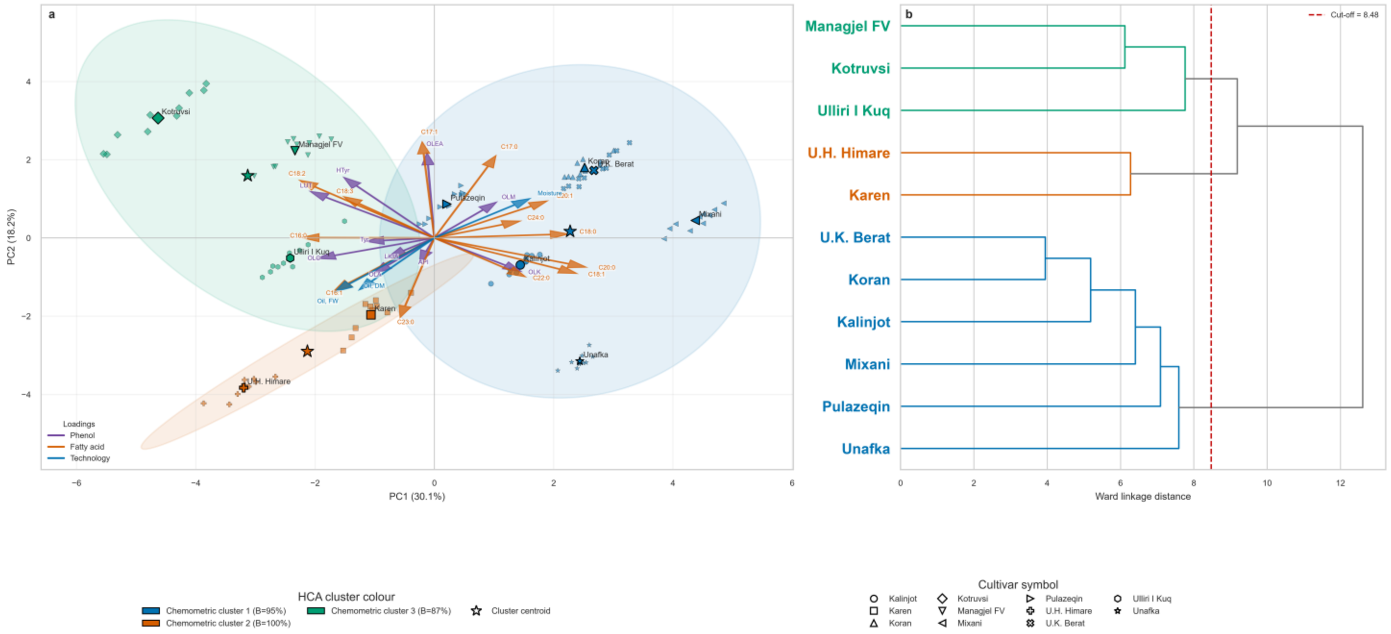

Figure S7. Joint chemical-technological chemometric cultivar clustering. (a) Principal component analysis score-loading biplot of the 10 phenolics, 13 fatty acids and 3 technological variables. Points represent independent sample-level oils; point symbols identify cultivars and colours identify the 3 joint hierarchical cluster analysis groups. Large cultivar symbols mark cultivar centroids, stars mark cluster centroids and shaded regions are 95% covariance ellipses. All 26 loading vectors are displayed and coloured by block: phenolics (purple), fatty acids (orange) and technological traits (blue). (b) Ward-linkage dendrogram of z-standardised cultivar means; the red dashed line shows the cut-off defining 3 clusters. Branch colours match panel (a). Abbreviations: PCA, principal component analysis; PC1, first principal component; PC2, second principal component; HCA, hierarchical cluster analysis; OLC, oleocanthal; OLA, oleacein; OLEA, oleuropein aglycone; OLM, oleomissional; LIGA, ligstroside aglycone; OLK, oleokoronal; HTyr, hydroxytyrosol; LUT, luteolin; API, apigenin; Tyr, tyrosol; C16:0, palmitic acid; C16:1, palmitoleic acid; C17:0, margaric acid; C17:1, margaroleic acid; C18:0, stearic acid; C18:1, oleic acid; C18:2, linoleic acid; C18:3, linolenic acid; C20:0, arachidic acid; C20:1, gadoleic acid; C22:0, behenic acid; C23:0, tricosanoic acid; C24:0, lignoceric acid; Oil_FW, oil content on a fresh-matter basis; Oil_DM, oil content on a dry-matter basis; FW, fresh-matter basis; DM, dry-matter basis; B, mean within-cluster bootstrap co-membership.

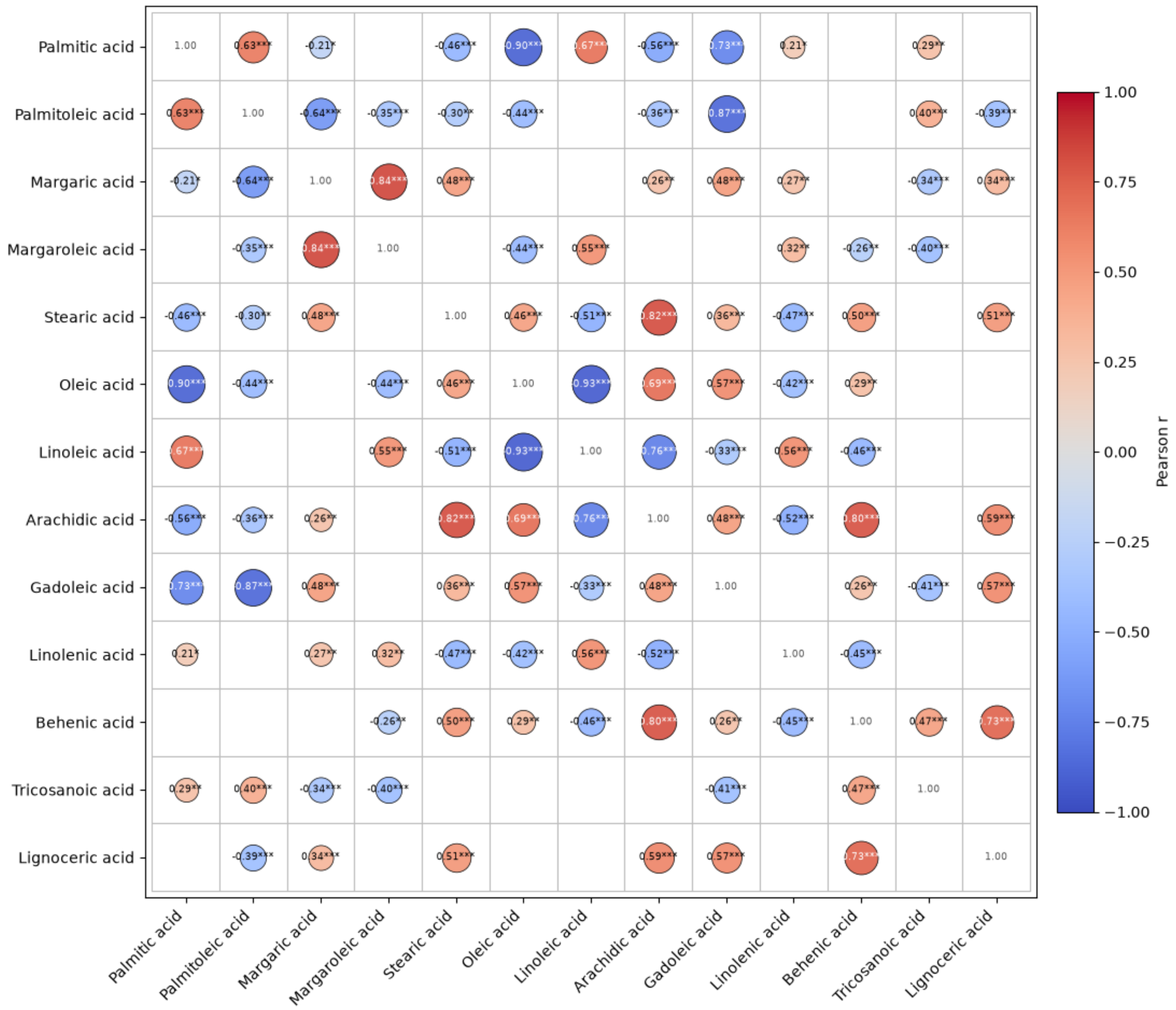

Figure S8. Exploratory Pearson correlation matrix among the 13 fatty-acid percentages across 110 independent sample-level oils. Circle colour indicates the sign of Pearson's correlation coefficient (r), circle size indicates the correlation scale in numerical level (r). Only associations that remained significant after Benjamini–Hochberg false-discovery-rate correction are displayed: * $q < 0.05$, ** $q < 0.01$ and *** $q < 0.001$; blank off-diagonal cells were not significant ($q \geq 0.05$), and diagonal cells equal 1.00.
