# Supplementary material for: Cultivar-Specific Phenolic and Fatty-Acid Composition of Albanian Monovarietal Olive Oils
Source: Foods. 2026 Oct 19;15(20):3726. doi: 10.3390/foods15203726 (PMC13704448; doi:10.3390/foods15203726)
Supplement: Supplementary file 1 [file foods-15-03726-s001.zip › Supplementary_Tables_Revised_v3. 08.09.26.pdf]

Article

Cultivar-specific phenolic and fatty-acid composition of Albanian monovarietal olive oils

Fatjon Hoxha[1†], Mivan Peçi[2†], Concepción Muñoz Díez[3], Feliciano Priego Capote[4], Adhurim Lazaj[5], Anila Kopali[1], Elisa Peçi[6], Onejda Kyçyk[7] and Hristofor Miho[3*]

Supplementary Tables

Table S1. Simple sequence repeat-authenticated cultivar designations and names used in the manuscript.

Cultivar manuscript designation name	Cultivar full name	Relevant synonym(s)
Kalinjot	–	–
Karen	–	–
Koran	–	–
Kotruvsi	–	Boçi
Managjel FV	Managjel Forme e Vecante	–
Mixani	–	Kushan
Pulazeqin	–	–
U.H. Himare	Ulli i Hollë i Himarës	Megaritiki
U.K. Berat	Ulli Kokërrmadh i Beratit	Chondrolia; Kalinjot Pobrat
Ulliri i Kuq	–	–
Unafka	–	–

Note: Simple sequence repeat (SSR) loci DCA3, DCA9, DCA11, DCA16, DCA18 and GAPU101 were used for true-to-type authentication and nomenclatural reconciliation only.

Table S2. Liquid chromatography–tandem mass spectrometry multiple-reaction-monitoring conditions used for phenolic determination.

Analyte	Retention time (min)	Precursor ion (m/z)	Product ion (m/z)	Tube lens (V)	Collision energy (eV)
**Hydroxytyrosol**	1.9	153.2	123.3	79	16
**Tyrosol**	2.2	137.1	119.3	55	17
**Oleacein**	3.2	319.7	69.5	89	32
**Oleomissional**	3.4	377.5	275.8	91	14
**Oleocanthal**	5.0	303.3	69.3	85	30
**Oleuropein aglycone**	5.6	377.5	275.8	91	14
**Oleokoronal**	5.6	361.6	291.7	78	17
**Luteolin**	5.7	284.8	133.3	104	43
**Naringenin (internal standard)**	6.0	271.1	119.1	92	20
**Apigenin**	6.3	269.1	117.1	103	42
**Ligstroside aglycone**	6.4	361.6	291.7	78	17

Note: Negative-ion multiple-reaction-monitoring mode. Oleomissional and oleokoronal were quantified through the response models of the corresponding closed monoaldehydic precursors, as stated in Materials and Methods. Abbreviations and units: min, minutes; m/z, mass-to-charge ratio; V, volts; eV, electronvolts.

Table S3. Matrix-matched calibration models used for absolute phenolic quantification.

Analyte	Calibration model	Coefficient of determination (R^2)
**Hydroxytyrosol**	y = (0.0772 ± 0.0021)x − (0.0842 ± 0.0356)	0.9866
**Tyrosol**	y = (0.0023 ± 0.0006)x − (0.0025 ± 0.0011)	0.9858
**Oleacein**	y = (0.0084 ± 0.0002)x − (0.0068 ± 0.0028)	0.9929
**Oleocanthal**	y = (0.0048 ± 0.0009)x − (0.0023 ± 0.0016)	0.9929
**Oleuropein aglycone**	y = (0.0828 ± 0.0019)x − (0.0592 ± 0.0335)	0.9896
**Ligstroside aglycone**	y = (0.3762 ± 0.0088)x − (0.2690 ± 0.1527)	0.9896
**Luteolin**	y = (0.1713 ± 0.0017)x − (0.0559 ± 0.0290)	0.9982

Analyte	Calibration model	Coefficient of determination (R^2)
**Apigenin**	y = (0.1042 ± 0.0012)x − (0.0142 ± 0.0200)	0.9975

Note: Calibration range: 0.8–40 mg kg^{-1}. Models were prepared in refined olive-pomace oil fortified with analytical standards. Abbreviation: R^2, coefficient of determination.

Table S4. One-way ANOVA testing the effect of cultivar on each analytical response.

Analytical group	Response	Transformation	df_1	df_2	F	p	η^2
Phenolic compounds	Oleocanthal	Square root	10	99	7076.84	p < 0.001	0.999
Phenolic compounds	Oleacein	log(1 + x)	10	99	431.51	p < 0.001	0.978
Phenolic compounds	Oleuropein aglycone	log(1 + x)	10	99	521.45	p < 0.001	0.981
Phenolic compounds	Oleomissional	log(1 + x)	10	99	357.01	p < 0.001	0.973
Phenolic compounds	Ligstroside aglycone	Square root	10	99	9842.63	p < 0.001	0.999
Phenolic compounds	Oleokoronal	log(1 + x)	10	99	230.85	p < 0.001	0.959
Phenolic compounds	Hydroxytyrosol	Original scale	10	99	959.81	p < 0.001	0.990
Phenolic compounds	Luteolin	log(1 + x)	10	99	1318.13	p < 0.001	0.993
Phenolic compounds	Apigenin	Yeo–Johnson (λ = -26.695)	10	99	13.35	p < 0.001	0.574
Phenolic compounds	Tyrosol	Original scale	10	99	514.94	p < 0.001	0.981
Phenolic compounds	Total quantified phenolics	log(1 + x)	10	99	1652.38	p < 0.001	0.994
Phenolic	EFSA-related mass sum	log(1 + x)	10	99	1404.73	p < 0.001	0.993

Analytical group	Response	Transformation	df_1	df_2	F	p	η^2
compounds							
Fatty acids	Palmitic acid	log(1 + x)	10	99	892.54	$p < 0.001$	0.989
Fatty acids	Palmitoleic acid	Original scale	10	99	3955.09	$p < 0.001$	0.998
Fatty acids	Margaric acid	Square root	10	99	834.49	$p < 0.001$	0.988
Fatty acids	Margaroleic acid	Yeo–Johnson ($\lambda = 8.870$)	10	99	870.35	$p < 0.001$	0.989
Fatty acids	Stearic acid	log(1 + x)	10	99	382.16	$p < 0.001$	0.975
Fatty acids	Oleic acid	Yeo–Johnson ($\lambda = 1.449$)	10	99	1462.61	$p < 0.001$	0.993
Fatty acids	Linoleic acid	Original scale	10	99	1273.33	$p < 0.001$	0.992
Fatty acids	Arachidic acid	Yeo–Johnson ($\lambda = -1.492$)	10	99	497.79	$p < 0.001$	0.980
Fatty acids	Gadoleic acid	Square root	10	99	585.61	$p < 0.001$	0.983
Fatty acids	Linolenic acid	Square root	10	99	393.96	$p < 0.001$	0.975
Fatty acids	Behenic acid	Yeo–Johnson ($\lambda = -21.736$)	10	99	381.80	$p < 0.001$	0.975
Fatty acids	Tricosanoic acid	Square root	10	99	529.53	$p < 0.001$	0.982
Fatty acids	Lignoceric acid	Square root	10	99	344.25	$p < 0.001$	0.972
Fatty acids	Oleic/linoleic ratio	Box–Cox ($\lambda = -0.447$)	10	99	1339.83	$p < 0.001$	0.993
Technological parameters	Fruit moisture	Yeo–Johnson ($\lambda = 2.894$)	10	99	12.16	$p < 0.001$	0.551
Technological parameters	Oil content, fresh-matter basis	log(1 + x)	10	99	16.25	$p < 0.001$	0.621
Technological parameters	Oil content, dry-matter basis	log(1 + x)	10	99	6.95	$p < 0.001$	0.412

Note: Each model included cultivar as a fixed factor and 110 oil samples observations (11 cultivars × 10 oil samples). df_1 and df_2 are the numerator and residual degrees of freedom, respectively. F and p values refer to the overall effect of cultivar tested by one-way ANOVA on the stated scale. η^2 was calculated as the between-cultivar sum of squares divided by the total sum of squares and is numerically equivalent to partial η^2 and model R^2 for these one-factor models.

Table S5. Cultivar membership and bootstrap support of the exploratory chemometric clusters.

Cultivar	Phenolic cluster	Bootstrap (%)	Fatty-acid cluster	Bootstrap (%)	Joint cluster	Bootstrap (%)
**Kalinjot**	2	100.0	1	88.6	1	95.0
**Karen**	4	99.9	2	100.0	2	99.8
**Koran**	2	100.0	1	88.6	1	95.0
**Kotruvsi**	3	99.9 (SR)	2	100.0	3	86.8
**Managjel FV**	4	99.9	2	100.0	3	86.8
**Mixani**	2	100.0	1	88.6	1	95.0
**Pulazeqin**	2	100.0	2	100.0	1	95.0
**U.H. Himare**	4	99.9	2	100.0	2	99.8
**U.K. Berat**	2	100.0	1	88.6	1	95.0
**Ulliri i Kuq**	1	100.0	2	100.0	3	86.8
**Unafka**	1	100.0	1	88.6	1	95.0

Note: Hierarchical cluster analysis was applied to z-standardised cultivar means using Euclidean distance and Ward's linkage. For clusters containing at least 2 cultivars, bootstrap support is the mean within-cluster pairwise co-membership over 1000 within-cultivar resamples. For the singleton phenolic cluster 3, 99.9 (SR) is the singleton-retention frequency: the percentage of bootstrap solutions in which 'Kotruvsi' remained a one-cultivar cluster. Pairwise co-membership and SR are complementary, but not identical, stability measures. The clusters are exploratory chemometric groupings and not genetic classifications.

Table S6. Silhouette comparison and bootstrap stability of the exploratory hierarchical cluster analysis solutions.

A. Silhouette comparison

Profile	Number of clusters	Mean silhouette width	Retained solution
**Phenolics**	2	0.251	No
**Phenolics**	3	0.294	No
**Phenolics**	4	0.291	Yes

Profile	Number of clusters	Mean silhouette width	Retained solution
**Phenolics**	5	0.295	No
**Fatty acids**	2	0.239	Yes
**Fatty acids**	3	0.180	No
**Fatty acids**	4	0.188	No
**Fatty acids**	5	0.166	No
**Joint profile**	2	0.172	No
**Joint profile**	3	0.179	Yes
**Joint profile**	4	0.171	No
**Joint profile**	5	0.124	No

B. Bootstrap stability

Profile	Cluster	Cultivars	n cultivars	Bootstrap support (%)
**Phenolics**	1	Ulliri i Kuq; Unafka	2	100.0
**Phenolics**	2	Kalinjot; Koran; Mixani; Pulazeqin; U.K. Berat	5	100.0
**Phenolics**	3	Kotruvsi	1	99.9 (SR)
**Phenolics**	4	Karen; Managjel FV; U.H. Himare	3	99.9
**Fatty acids**	1	Kalinjot; Koran; Mixani; U.K. Berat; Unafka	5	88.6
**Fatty acids**	2	Karen; Kotruvsi; Managjel FV; Pulazeqin; U.H. Himare; Ulliri i Kuq	6	100.0
**Joint profile**	1	Kalinjot; Koran; Mixani; Pulazeqin; U.K. Berat; Unafka	6	95.0
**Joint profile**	2	Karen; U.H. Himare	2	99.8
**Joint profile**	3	Kotruvsi; Managjel FV; Ulliri i Kuq	3	86.8

Note: All variables were z-standardised before hierarchical cluster analysis. Silhouette values compare solutions containing 2–5 clusters. The phenolic solution was retained at Ward distance 5 (4 clusters); the fatty-acid and joint solutions retained 2 and 3 clusters, respectively. For clusters containing at least 2 cultivars, bootstrap support is mean within-cluster pairwise co-membership over 1000 resamples. For the singleton phenolic cluster 3, SR denotes singleton-retention frequency; 'Kotruvsi' remained alone in 999 of 1000 bootstrap solutions (99.9%). Abbreviations: n, number of cultivars; SR, singleton-retention frequency.

Table S7. PCA loadings and variable contributions to the 2 displayed principal components for (A) phenolics, (B) fatty acids and (C) the joint chemical-technological matrix.

A. Phenolic PCA (PC1, 29.5%; PC2, 26.9%)

Variable	PC1 loading	PC1 contribution (%)	PC2 loading	PC2 contribution (%)
Oleocanthal	0.575	11.1	-0.560	11.6
Oleacein	-0.176	1.0	-0.466	8.0
Oleuropein aglycone	-0.296	2.9	-0.598	13.2
Oleomissional	-0.813	22.2	-0.159	0.9
Ligstroside aglycone	0.610	12.5	0.574	12.2
Oleokoronal	-0.155	0.8	0.853	26.8
Hydroxytyrosol	0.447	6.7	-0.482	8.6
Luteolin	0.706	16.7	-0.550	11.2
Apigenin	0.056	0.1	-0.250	2.3
Tyrosol	0.880	26.0	0.379	5.3

B. Fatty-acid PCA (PC1, 41.1%; PC2, 24.7%)

Variable	PC1 loading	PC1 contribution (%)	PC2 loading	PC2 contribution (%)
Palmitic acid	-0.787	11.5	-0.112	0.4
Palmitoleic acid	-0.618	7.1	-0.642	12.7
Margaric acid	0.310	1.8	0.845	22.0
Margaroleic acid	-0.168	0.5	0.846	22.1
Stearic acid	0.764	10.8	0.088	0.2
Oleic acid	0.838	13.0	-0.238	1.7
Linoleic acid	-0.787	11.5	0.481	7.1
Arachidic acid	0.922	15.8	-0.192	1.1
Gadoleic acid	0.736	10.0	0.491	7.4
Linolenic acid	-0.462	3.9	0.522	8.4
Behenic acid	0.627	7.3	-0.333	3.4
Tricosanoic acid	-0.100	0.2	-0.632	12.3
Lignoceric acid	0.597	6.6	0.181	1.0

C. Joint PCA (PC1, 30.1%; PC2, 18.2%)

Variable	PC1 loading	PC1 contribution (%)	PC2 loading	PC2 contribution (%)
Oleocanthal	-0.682	5.9	-0.182	0.7
Oleacein	-0.352	1.6	-0.315	2.1
Oleuropein aglycone	-0.043	0.0	0.754	11.9
Oleomissional	0.363	1.7	0.319	2.1

Variable	PC1 loading	PC1 contribution (%)	PC2 loading	PC2 contribution (%)
Ligstroside aglycone	-0.269	0.9	-0.169	0.6
Oleokoronal	0.505	3.2	-0.297	1.8
Hydroxytyrosol	-0.527	3.5	0.538	6.1
Luteolin	-0.722	6.6	0.413	3.6
Apigenin	-0.086	0.1	-0.215	1.0
Tyrosol	-0.400	2.0	-0.030	0.0
Palmitic acid	-0.777	7.6	0.003	0.0
Palmitoleic acid	-0.583	4.3	-0.468	4.6
Margaric acid	0.358	1.6	0.731	11.2
Margaroleic acid	-0.071	0.1	0.853	15.2
Stearic acid	0.781	7.7	0.035	0.0
Oleic acid	0.828	8.7	-0.315	2.1
Linoleic acid	-0.784	7.8	0.511	5.5
Arachidic acid	0.883	9.9	-0.260	1.4
Gadoleic acid	0.657	5.5	0.329	2.3
Linolenic acid	-0.527	3.5	0.366	2.8
Behenic acid	0.530	3.6	-0.345	2.5
Tricosanoic acid	-0.199	0.5	-0.707	10.4
Lignoceric acid	0.500	3.2	0.150	0.5
Moisture	0.556	3.9	0.350	2.6
Fresh Matter Oil Content	-0.578	4.2	-0.477	4.8
Dry Matter Oil Content	-0.438	2.4	-0.461	4.5

Note: Loadings are correlations between each z-standardised variable and the component score. Contribution (%) was calculated within each component as the squared loading divided by the sum of squared loadings × 100. Abbreviations: PCA, principal component analysis; PC1, first principal component; PC2, second principal component.
